# Structure, mineralogy, and microbial diversity of geothermal spring microbialites associated with a deep oil drilling in Romania

**DOI:** 10.3389/fmicb.2015.00253

**Published:** 2015-03-30

**Authors:** Cristian Coman, Cecilia M. Chiriac, Michael S. Robeson, Corina Ionescu, Nicolae Dragos, Lucian Barbu-Tudoran, Adrian-Ştefan Andrei, Horia L. Banciu, Cosmin Sicora, Mircea Podar

**Affiliations:** ^1^Taxonomy and Ecology, Algology, National Institute of Research and Development for Biological Sciences, Institute of Biological ResearchCluj-Napoca, Romania; ^2^Molecular Biology and Biotechnology Department, Faculty of Biology and Geology, Babeş-Bolyai UniversityCluj-Napoca, Romania; ^3^Biosciences Division, Oak Ridge National LaboratoryOak Ridge, TN, USA; ^4^Fish, Wildlife, and Conservation Biology, Colorado State UniversityFort Collins, CO, USA; ^5^Geology Department, Faculty of Biology and Geology, Babeş-Bolyai UniversityCluj-Napoca, Romania; ^6^Kazan (Volga Region) Federal UniversityTatarstan, Russia; ^7^Electron Microscopy Center, Faculty of Biology and Geology, Babeş-Bolyai UniversityCluj-Napoca, Romania; ^8^Molecular Biology Center, Institute for Interdisciplinary Research on Bio-Nano-Sciences, Babeş-Bolyai UniversityCluj-Napoca, Romania; ^9^Biological Research CenterJibou, Romania

**Keywords:** hot springs, carbonate, biomineralization, amplicon sequencing, microbial diversity, oil drill

## Abstract

Modern mineral deposits play an important role in evolutionary studies by providing clues to the formation of ancient lithified microbial communities. Here we report the presence of microbialite-forming microbial mats in different microenvironments at 32°C, 49°C, and 65°C around the geothermal spring from an abandoned oil drill in Ciocaia, Romania. The mineralogy and the macro- and microstructure of the microbialites were investigated, together with their microbial diversity based on a 16S rRNA gene amplicon sequencing approach. The calcium carbonate is deposited mainly in the form of calcite. At 32°C and 49°C, the microbialites show a laminated structure with visible microbial mat-carbonate crystal interactions. At 65°C, the mineral deposit is clotted, without obvious organic residues. Partial 16S rRNA gene amplicon sequencing showed that the relative abundance of the phylum *Archaea* was low at 32°C (<0.5%) but increased significantly at 65°C (36%). The bacterial diversity was either similar to other microbialites described in literature (the 32°C sample) or displayed a specific combination of phyla and classes (the 49°C and 65°C samples). Bacterial taxa were distributed among 39 phyla, out of which 14 had inferred abundances >1%. The dominant bacterial groups at 32°C were *Cyanobacteria, Gammaproteobacteria, Firmicutes, Bacteroidetes, Chloroflexi, Thermi, Actinobacteria, Planctomycetes*, and *Defferibacteres*. At 49°C, there was a striking dominance of the *Gammaproteobacteria*, followed by *Firmicutes, Bacteroidetes*, and *Armantimonadetes*. The 65°C sample was dominated by *Betaproteobacteria, Firmicutes*, [OP1], *Defferibacteres, Thermi, Thermotogae*, [EM3], and *Nitrospirae*. Several groups from *Proteobacteria* and *Firmicutes*, together with *Halobacteria* and *Melainabacteria* were described for the first time in calcium carbonate deposits. Overall, the spring from Ciocaia emerges as a valuable site to probe microbes-minerals interrelationships along thermal and geochemical gradients.

## Introduction

Contemporary microbial mats provide insight into ancient microbial life and the processes of biomineralization that led to the formation of some sedimentary rocks. Lithifying microbial mats facilitate trapping and binding of sediments and precipitation of minerals into sedimentary structures known as microbialites. Fossil lithified microbial mats have been studied intensively and have been linked to early life on Earth. Evidence from Archaean deposits suggests that the evolution of cyanobacteria or other filamentous bacteria started at least 2.9 billion years (Ga) ago, possibly even 3.5 Ga (Walsh, 1992; Schopf, 2002; Furnes et al., 2004; Tice and Lowe, 2004; Westall et al., 2006; Noffke et al., 2008). Structures from the Pilbara Craton (Western Australia) consist of nodular, wavy-laminated and coniform stromatolites that have been described from chert units within the ~3.49 Ga Dresser and ~3.43 Ga Strelley Pool Formations (Walter et al., 1980; Hofmann et al., 1999; Allwood et al., 2006). The biogenic nature of those structures, inferred largely upon simple macro-morphological comparisons with modern-day structures (Schopf, 2006), has been questioned (Brasier et al., 2006; McLoughlin et al., 2008).

Descriptions of the biological attributes and biodiversity of modern lithified microbial mats is important to better understand the mechanisms behind the genesis of ancient lithified microbial mats and the associated microbial diversity and functional traits that may have influenced their formation. Modern deposits have been described from a wide variety of environments, including hot springs (Berelson et al., 2011; Pepe-Ranney et al., 2012), hypersaline and alkaline lakes (Dupraz and Visscher, 2005; Couradeau et al., 2011; Schneider et al., 2013), freshwater environments (Breitbart et al., 2009; Santos et al., 2010; Centeno et al., 2012; Nitti et al., 2012; Farias et al., 2013, 2014), and marine and coastal environments (Reid et al., 2000; Baumgartner et al., 2009; Goh et al., 2009; Khodadad and Foster, 2012).

**Figure 1:**
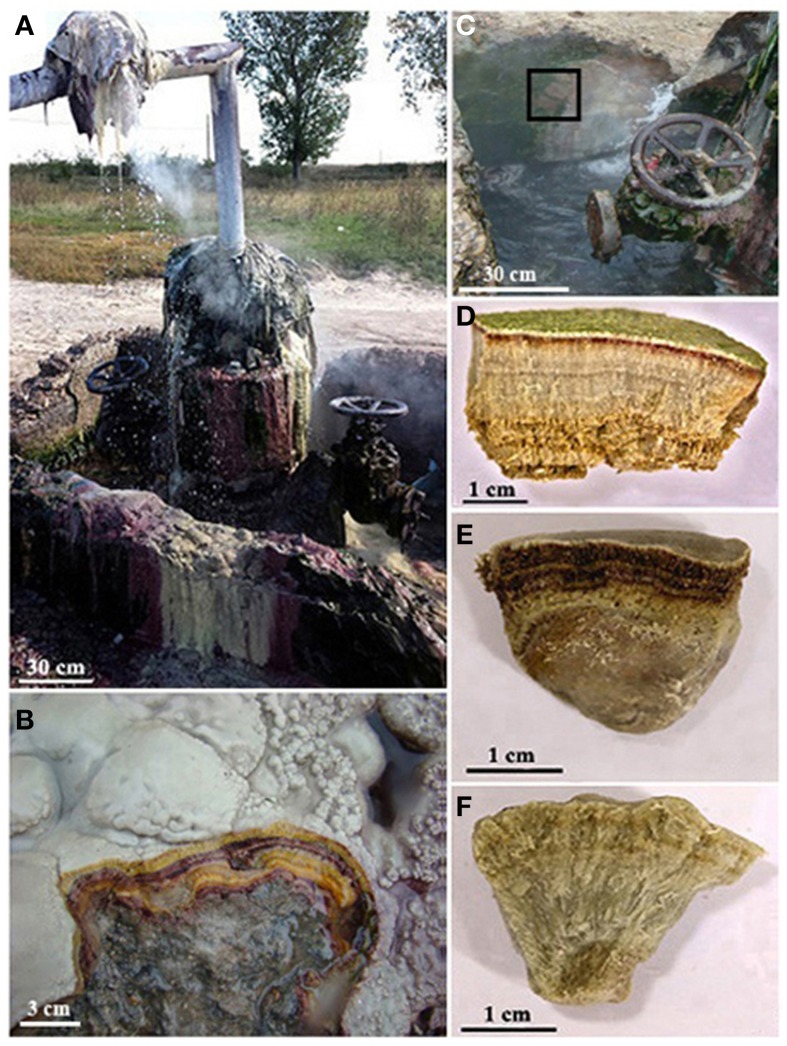
**(A)** General view of the drilling from Ciocaia; **(B)** Layered fine strata forming mammillary coatings at 49°C, seen in fresh break; **(C)** The small pool formed where the hot water comes out; the rectangle marks the 32°C sampling area, near the exit of thermal water, but not in direct contact. **(D)** Detailed view of 32°C mineral deposit. **(E)** Detailed view of 49°C mineral deposit. **(F)** Detailed view of 65°C mineral deposit.

This study is the first to describe the structure and diversity of mesophilic-to-thermophilic microbial mat communities associated with mineral deposits from an abandoned oil well drill site in Ciocaia, Romania (N 47°20.471′; E 22°02.744′) (Figure 1). Since 1960, hundreds of wells were drilled in this region in search of oil and hot water (Bendea et al., 2013). There is a continuous flow of hot water from some abandoned wells that has led to the formation of microbial mats and crystalline and microcrystalline carbonates, is in the case of the well from Ciocaia that was abandoned in the mid-1970s. This particular site was chosen for investigation due to its elevated carbonate concentrations in the fluids relative to other wells in the area (Coman et al., 2011, 2012, 2013) that may facilitate carbonate precipitation. The aims of this paper are: (i) to investigate the composition and abundance of minerals in the microbialites from Ciocaia, together with the description of their macro- and microstructure, that may provide information regarding their biogenic nature; (ii) to describe the microbial diversity in the carbonate deposits and to discuss the putative metabolic diversity in the microbial communities, that may influence the process of carbonate mineralization.

## Materials and methods

### Description of mineral deposits and sampling

Three sedimentary structures, each set in a specific microenvironment, were identified (Table 1) and sampled in April 2013: (a) A fibrous crystalline mineral deposit, with a colored multi-layer appearance that ranged from green at the top to red and light orange toward the bottom (Figure 1D) (sample code C32). The deposit was not in direct contact with the water flow but occasionally hydrated by water splashes from the surrounding structures (Figure 1C) and had a surface temperature of 32°C; (b) A mineral deposit forming mammillary crusts (Figure 1B) also with a layered structure, marked by the combination of red and yellow layers (Figures 1B,E) (sample code C49). The geothermal water was flowing over at a rate of ~0.1 l·min^−1^(Figure 1B), maintaining a temperature of 49°C at the surface of the deposit; (c) A relatively homogeneously creamy-colored mineral deposit with a fibrous crystalline structure (Figure 1F) (sample code C65). This deposit is permanently submerged at 65°C. Each sample was analyzed by polarized light and scanning electron microscopy, together with description of mineralogy and microbial diversity.

**Table 1:**
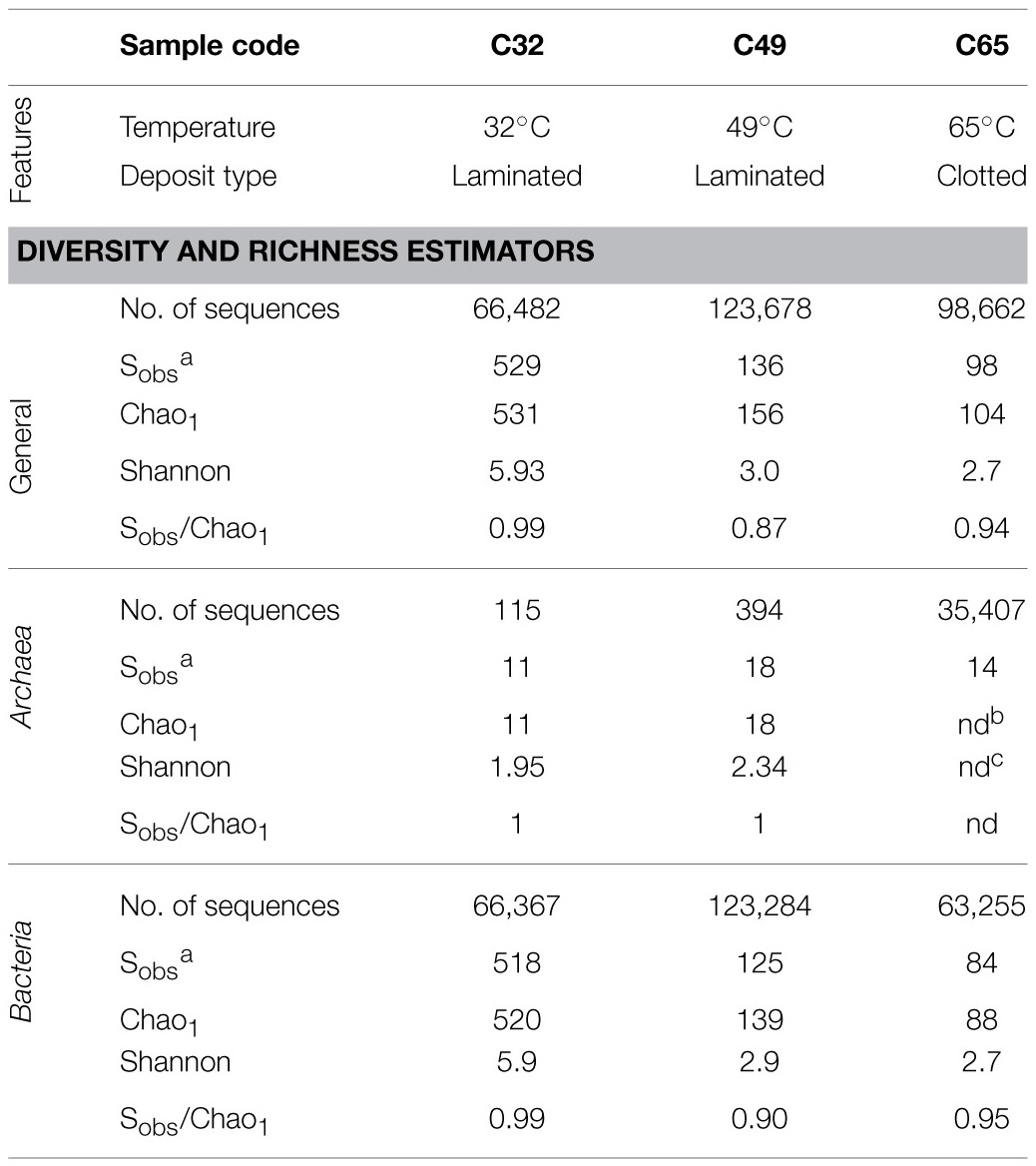
**Diversity and richness estimators for sequencing libraries**.

*^a^Observed richness*.

*^b^Not determined due to lack of doubletons*.

*^c^Not determined due to an almost absolute dominance of a single OTU (equitability index = 0.01)*.

For SEM and mineralogical analyses, ~5 cm^3^ of sample were collected in duplicate using sterile instruments and placed into sterile Petri dishes. For microbial DNA extraction, ~3 cm^3^ of mineral deposit were placed into sterile 50 ml Falcon tubes and immediately frozen in liquid nitrogen. Mineralogical observations were performed on thin sections obtained using a diamond saw.

### Polarized light microscopy and scanning electron microscopy

Micro-scale observations were made using a Nikon TE-2000 petrographic microscope equipped with a Nikon D90 digital camera (Nikon Inc., Melville, NY, USA). For SEM, the samples were fractured in liquid nitrogen, fixed on copper holders, covered with a 10 nm gold layer and observed with a JEOL JSM 5510LV electron microscope (JEOL, Tokyo, Japan).

### Mineralogical analysis using X-ray diffraction analysis (XRD)

The mineral composition was determined by X-ray powder diffraction using a Shimadzu 6000 diffractometer with CuKα radiation and Ni filter. The samples were hand milled in an agate mortar and measured from 2 to 60°2θ, with a scan speed of 2°/min.

### DNA extraction

Several published methods and commercial kits for DNA extraction were employed, but without the expected result (i.e., high molecular weight genomic DNA suitable for PCR amplification of 16S rRNA genes) (data not shown). Thus, a DNA extraction protocol was adapted after Reyes-Escogido et al. (2010) that combines the use of a chelating agent (Chelex-100, Bio-Rad, USA) and microwave radiation. DNA was extracted in triplicate and pooled. To remove the calcium carbonate, ~2 cm^3^ from each deposit was finely grinded using sterile mortars and pestles in a laminar flow hood. The resulting material was transferred to a sterile plastic tube containing 0.5 M Na_2_EDTA and mixed on an orbital shaker for 30 min at 37°C. After centrifugation for 5 min at 8000 × g, the supernatant was discarded and the Na_2_EDTA step was repeated twice. The sediment was resuspended in 1 ml TE buffer (10 mM Tris Base, 1 mM Na_2_EDTA, pH 7.5). Each sample (800 μl) was transferred to sterile 1.5 ml plastic microfuge tubes and spun for 5 min at 8000 × g and 4°C. Cells were resuspended in 50 μl of TES buffer (10mM TRIS Base, 1 mM Na_2_EDTA, 0.5% SDS, pH 7.5). The tubes were irradiated in a microwave oven for 30 sat 625 W, then 150 μg of proteinase K and 20 μg of RNase A were immediately added, followed by another round of microwave irradiation. The samples were incubated at room temperature for 2 min, then 150 μl of TE buffer containing 25 mg Chelex 100 (Bio-Rad, USA) were added, followed by another round of microwave irradiation. The samples were spun for 5 min at 12,000 × g and 4°C. The supernatant was collected and purified using the phenol/chloroform/isoamyl alcohol protocol (Moore, 1995). The quality and quantity of DNA was assessed by electrophoresis on a 1% agarose gel (data not shown).

### Real-time quantitative PCR (RT-qPCR) and data analysis

The absolute quantification of archaeal and bacterial 16S rRNA gene copies was performed using the SsoFast Eva Green Supermix (Bio-Rad, Hercules, CA, USA) and 16S rRNA gene group-specific primers (see Supplementary Material) on an iCycler IQ5 Real-Time System (Bio-Rad, Hercules, CA, USA). For archaeal 16S rRNA gene amplification reactions were carried out in triplicate and the reaction mixture contained the following components: 7 μl 1 × Sso Fast EvaGreen SuperMix (Bio-Rad, Hercules, CA, USA), 0.4 μM of the forward 931F and reverse M1100R primers, 10 ng of DNA and RNase/DNase-free water to a final volume of 14 μl. The reactions were carried out as follows: 180 s initial denaturation at 98°C, followed by 45 cycles of: 25 s denaturation at 98°C, 25 s primer annealing at 61.5°C, and 30 s extension at 72°C. For assessing the specificity of the primers a post-PCR melting curve analysis was performed, in which the temperature varied between 60°C and 90°C in 0.5°C increments with subsequent plate readings. The same reaction components and protocol were used for the bacterial RT-qPCR with the following modifications: the primers used were 338F and 518R, and their annealing temperature was 61°C.

The absolute 16S rRNA genes numbers were estimated based on standard curves that contained serial 10-fold dilutions of genomic DNA from *Halobacterium salinarum* DSM 3754 and *Escherichia coli* K-12 subspecies DH10B (Invitrogen, Carlsbad, CA, USA). Reaction efficiencies and data analyses were assessed using the background subtracted data and the LinRegPCR software. The numbers of cells from the numbers of 16S rRNA genes found in the samples was inferred taking into account the variation in ribosomal RNA operons by using a mean of 4.02 for Bacteria and 1.63 for Archaea (rrnDB version 4.3.3) (Stoddard et al., 2014).

### Sequencing, quality control, and analysis of illumina sequencing data

Variable region V4 of the 16S rRNA gene was amplified using the Archaea/Bacteria universal primer pair 515F-806R following the protocol of Lundberg et al. (2013). The primer pair was selected as it exhibits few biases against individual bacterial taxa (Liu et al., 2007; Fierer et al., 2012). In brief, two step single strand reactions with diversity generation primers were performed followed by the PCR with the barcode primers (full sequence of primers is provided in the Supplementary Material). Each sample was amplified in triplicate, combined, and cleaned using the Qiagen PCR clean up kit. A composite sample for sequencing was created by combining equimolar ratios of amplicons from the individual samples and multiplex paired-end sequencing of barcoded amplicons was performed on a MiSeq machine (Illumina Inc., San Diego, CA, USA) at the Oak Ridge National Laboratory (Oak Ridge, TN, USA). Raw sequencing data was deposited in the GenBank SRA database (National Center for Biotechnology Information—NCBI) under the Accession Number SRP043549. Sequence data was processed and quality-controlled through a combination of the UPARSE and QIIME pipelines (Caporaso et al., 2010a; Edgar, 2013). Cutadapt (Martin, 2011) was used in paired-end mode to trim sequencing primers from the forward and reverse reads. Read pairs were discarded if the either the forward or reverse primer was not detected. Paired-ends were merged using the “-fastq_mergepairs” option of usearch (v.7.0.1001; Edgar, 2013). An in-house python script was used to remove unused barcodes of paired-end sequences that did not survive merging. The QIIME (v1.7; Caporaso et al., 2010a) script, split_libraries_fastq.py, was used to demultiplex the sequence data with the quality filter set to zero. Remaining quality control processing was carried out via the UPARSE pipeline (Edgar, 2013) including de novo and reference-based chimera detection. The resulting OTU table was converted to BIOM format (McDonald et al., 2012). Taxonomy was assigned using the RDP classifier (Wang et al., 2007) against the updated May 2013 “13_5/13_8” Greengenes database (DeSantis et al., 2006; McDonald et al., 2012; Werner et al., 2012) via QIIME (Caporaso et al., 2010a). A phylogeny was constructed using FastTree (Price et al., 2010) from a masked PyNAST (Caporaso et al., 2010b) alignment. The resulting phylogeny was manually rooted to Archaea via Dendroscope (v3; Huson and Scornavacca, 2012). Finally, various diversity metrics were calculated via QIIME (Caporaso et al., 2010a).

Finally, we compared the microbial diversity in the Ciocaia samples with existing microbial mat/microbialite studies from the following GenBank SRRs: SRR329490 (Farias et al., 2013); SRR627689, SRR627690, SRR627691, SRR627395 (Farias et al., 2014); SRR961678, SRR952918, SRR952917, SRR952915, SRR952913 (Rasuk et al., 2014); SRR350006 (Centeno et al., 2012). As several of these studies used a variety of different DNA extraction and sequencing protocols and/or target a different variable region of the 16S rRNA gene, we performed closed-reference OTU picking via QIIME (Caporaso et al., 2010a) at 97% sequence similarity; after quality filtering and trimming via UPARSE (Edgar, 2013). Closed-reference OTU picking enables the comparison of these different studies by only retaining those sequences that match a full-length representative sequence within the GreenGenes reference database (McDonald et al., 2012; Werner et al., 2012).

### Phylogenetic analysis

As a significant amount of archaeal taxa could not be accurately classified using solely the Greengenes database, the phylogenetic placement of OTUs relative to known curated sequence data was determined by aligning the representative OTU sequences with SINA (Pruesse et al., 2012) and then importing the alignment into the ARB software package (Ludwig et al., 2004). The sequences were inserted into the SILVA SSU Ref database (v115) with domain-specific position variable filters via parsimony insertion. As ~30% of the *Archaea* partial 16S rRNA gene sequences from Ciocaia were clustered as “uncultured archaeon,” the phylogenetic analysis was continued with a second approach by manually selecting 16S rRNA gene sequences deposited in the GenBank database (NCBI). Multiple sequence alignments were performed with ClustalW in MEGA5 (Tamura et al., 2011). JModelTest program (Guindon and Gascuel, 2003; Posada, 2003) was used to select an appropriate model of sequence evolution for phylogenetic inference. A maximum likelihood tree was constructed with MEGA version 5 (Tamura et al., 2011) under the best model (Tamura-Nei), with 500 bootstrap replicates. As the phylum *Nanoarchaeota* is a more recent ancestor of *Euryarchaeota* and *Crenarchaeota, Nanoarchaeum equitans* was chosen as outgroup.

## Results and discussion

### Geologic background and mineralogy of the carbonate deposits

The lithological column from Ciocaia consists mainly of carbonate-rich rocks and marly limestones. Between the surface (105 m above sea level) and the depth of 120 m, the well crossed Quaternary sand and gravel with mudstone and silt intercalations. The underlying Upper Pannonian sediments have around 1300 m thickness and were encountered down to 1420 m depth. They consist of sands with calcareous mudstone intercalations or mudstone and calcareous mudstone with sandy intercalations. Between 1420 and 2320 m Lower Pannonian calcareous mudstone contains thin layers of sandstone. The sediments spanning between 2320 and 2434 m depth are assigned to Sarmatian. Beneath the Sarmatian sediments until the final depth of 2536 m, the drill crossed biotite and garnet gneiss of the basement of the area (data courtesy of SC Transgex Oradea). The effluent geothermal water has a temperature of 65°C and a high HCO^−^_3_concentration of ~7.3 mg·l^−1^ (Ţenu et al., 1981; Romanian Waters Administration, personal communication).

Macroscopically, the carbonate crusts show an overall light cream color, with a visible fibrous structure. Dark thin layers (from <1 mm up to few mm thickness), parallel with the substrate (base of the deposit) gives a banded, rhythmical appearance in the case of C32 and C49. The X-ray diffraction data are similar for all the samples investigated and indicate the presence of mostly calcite, accompanied by some clay minerals (probably illite and smectite). Very weak lines, which may be tentatively assigned to aragonite, are present.

**Figure 2:**
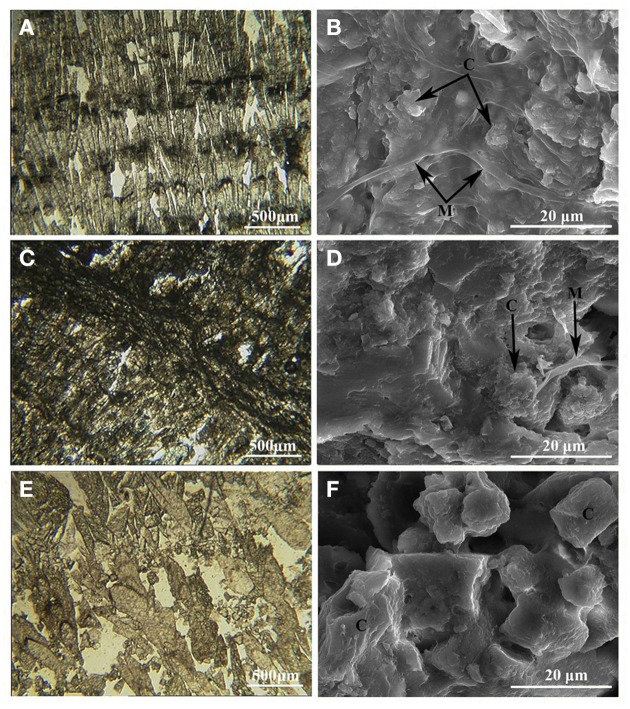
**Polarized light (one polarizer) microphotos of carbonate crusts (A,C,E) and SEM detail view of intermediate dark layers (B,D,F)**. **(A)** 32°C carbonate deposit (sample C32) displaying a laminated pattern of crystals arranged in a succession of lighter and darker laminae. **(B)** Detailed view of the darker layer in C32 displaying a close association between the crystals and organic material. **(C)** 49°C carbonate deposit (sample C49) where the dark laminae are found between well-defined layers of crystallized calcite. **(D)** Detailed view of the darker layer in C49 displaying a close association between the crystals and organic material. **(E)** 65°C carbonate deposit (sample C65) displaying a more irregular pattern of crystal growth with no intermediate black layers. **(F)** Detailed view of sample C65, showing carbonate crystals without an association with a well-defined microbial mat. M: microbial cells embedded in EPS; C, carbonate crystal.

Polarized light microscopy reveals that the carbonate samples from Ciocaia consist of bundles of elongated scalenohedral crystals displaying slight radial orientation (Figures 2A,C,E), with a more or less regular lamination of darker and lighter layers. In C32 and C49, the pattern of dark stripes occurs within carbonate crystals (Figures 2A,C). In C65, the crystal growth is more irregular and no dark layers were observed (Figure 2E). The darker layers are built of micritic carbonate and while in the case of C32 and C49 they are visibly associated with organic material (Figures 2B,D), in the C65 sample this could not be observed (Figure 2F). These organic-rich layers are usually thin compared with the thicker lighter layers. The lamination pattern can be best observed at 32°C. It starts to fade away with the increase of temperature and the lack of organic material, the precipitation of minerals being random at 65°C.

### Microbial mat-carbonate crystal interactions

**Figure 3:**
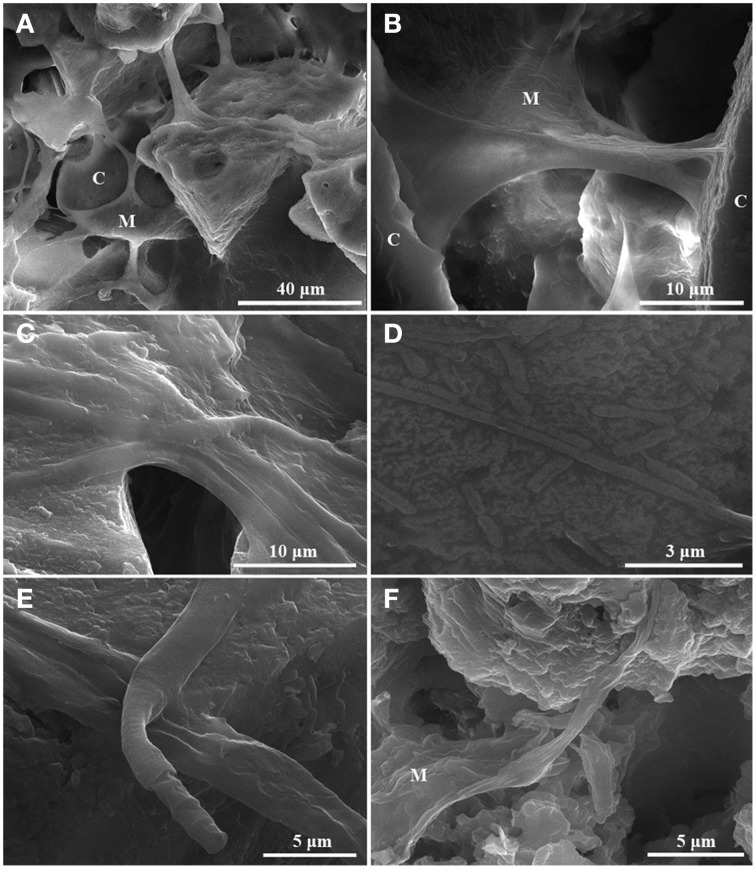
**Scanning electron microscopy images presenting the microbial mat-crystals interaction in the carbonate deposits from Ciocaia**. **(A)** The microbial mat traps and binds the crystals (C32). **(B)** The microbial mats form connecting bridges between crystals (observed in C49 and rarely in C65). **(C,D)** The microbial mat or the individual bacterial filaments or rods colonizing the mineral deposit (**C**—C32; **D**—C65). **(E,F)** Individual filaments (**E**—C32), as well as the entire microbial biofilms (**F**—C49) are included in what appears to be a thick mass of extracellular polymeric substances. M, microbial mat; C, carbonate crystal.

Several types of microbial mat-carbonate crystals interactions were observed using SEM: (i) trapping and binding of crystals (Figure 3A), a key interaction involved in the genesis of lithified communities (Dupraz et al., 2009); (ii) microbial mats forming connecting bridges between crystals (Figures 3A,B); (iii) microbial mat and individual bacterial filaments or rods colonizing the mineral mass (Figures 3C,D). The bacterial filaments observed are 0.5–2.5 μm in diameter and tens of μm in length (Figures 3C,D). Sometimes, cyanobacteria form a fibrous net (Figure 3D), a similar type of structure being described in the 3.446 Ga “Kitty's Gap Chert,” Warrawoona Group, Pilbara by Westall et al. (2006). The individual filaments (Figure 3E), as well as the entire microbial biofilms (Figure 3F) are included in what appears to be a thick mass of extracellular polymeric substances (EPS), which may have an important role in carbonate precipitation (Dupraz et al., 2009). Laboratory studies show that the particles precipitate in the organic matrix of the EPS; the activity of heterotrophic microbes can initiate micro-domains that act as nucleation sites for carbonate precipitation (Kawaguchi and Decho, 2002a,b; Braissant et al., 2007). Usually, when it comes to individual filaments the EPS can be subdivided into 2 areas: an inner one, directly in contact with the cell and an outer one, at the exterior. The inner area of the EPS is used as a defense mechanism (Obst et al., 2009) because it accumulates Ca^2+^ more heavily than the outer area and carbonate precipitates in the form of aragonite. The aragonite is unstable and it dissolves when the oversaturation of the sorrounding environment dissapears. If the oversaturation period persists, CaCO_3_ would be deposited in the form of calcite, which is more stable and does not dissolve when the oversaturation drops.

### Microbial abundance and diversity

Bacterial and archaeal cell concentrations were estimated from qPCR data. *Bacteria* dominated the microbial community in C32 (2.1 ± 0.01 × 10^8^ cells/g wet weight; 98.6% of total cell numbers) and C49 (7.2 ± 0.01 × 10^8^ prokaryotic cells/g of carbonate; 99.8% of total prokaryotic cell numbers). In C65, bacterial cells accounted for 54.6% of total prokaryotic cell numbers (5.7 ± 0.02 × 10^7^ prokaryotic cells/g of carbonate). *Archaea* represented less than 2% in the C32 and C49 samples (2.9 ± 0.01 × 10^6^ prokaryotic cells/g of carbonate—1.34% and 1.0 ± 0.01 × 10^6^ prokaryotic cells/g of carbonate—0.2%, respectively) and were in almost equal amounts with *Bacteria* in C65 (4.7 ± 0.03 × 10^7^ prokaryotic cells/g of carbonate—45.4%).

### Microbial taxon richness and diversity coverage

Quality filtration of the raw sequencing data resulted in a total of 288,822 sequences with an average read length of 254 bp. Each sample consisted of 66,500–123,000 sequencing reads, which were clustered into OTUs at 97% sequence identity. Of these, 349 sequences could not be assigned to any particular phylum (316 sequences for C32, 17 for C49 and 16 for C65). The rarefaction curve constructed for the C32 sample exhibited a steeper slope than those determined for the C49 and C65 samples, demonstrating a greater microbial richness at lower temperature (Supplementary Figure S3). This observation is sustained by the Chao1 index, which was calculated to be 531 for the C32 sample, while the corresponding values for the C49 and C65 samples were 156 and 104, respectively (Table 1). Shannon,s index was similar for C49 and C65 samples (3.0 and 2.7), but doubled in value for the C32 sample (5.94) (Table 1). As the three rarefaction curves reached saturation (Supplementary Figure S3), it was considered that the sequencing data is reliable for an accurate characterization of prokaryotic diversity in the investigated samples. For an easier overview of microbial diversity in the C32, C49, and C65 samples a Krona chart was generated (see Supplementary Material) (Ondov et al., 2011) using the SILVAngs web application and the ARB-SILVA database. Even though there are slight differences in the abundance percentages when compared to the Greengenes database (used by QIIME), the overall microbial taxonomy of Ciocaia samples generated with both pipelines is similar. Thus, the data generated with QIIME will be presented and discussed further on.

### Archaeal diversity and community structure

After quality filtering, 35,916 archaeal 16S rRNA partial gene sequences were obtained. Their abundances in the C32 and C49 libraries are low, representing only 0.17 and 0.31%, respectively. The percentage of archaeal sequences increased dramatically in the C65, counting for 35.88% of the entire library. Clustering at 97% cut-off shows the existence of 11 OTUs in C32 library, 18 OTUs in C49, and 14 OTUs in C65 (Table 1). Even though the biodiversity of Archaea in these samples is low, it is comparable or even higher than that of other marine or freshwater carbonate deposits (Goh et al., 2009; Couradeau et al., 2011; Mobberley et al., 2012).

For a better characterization of the archaeal diversity, two phylogenetic trees were constructed: one that uses known curated sequences from the SILVA SSU Ref database in the ARB software package and a simplified phylogenetic tree constructed using manually picked sequences deposited in the GenBank database, in the MEGA 5 software package. They were named “ARB tree” (Supplementary Figure S1) and “MEGA tree” (Supplementary Figure S2).

Within the *Archaea* domain, the phylum *Euryarchaeota* dominated the three samples almost completely, with a distribution ranging between 92 and 99.8%, while the *Crenarchaeota* phylum was identified only in the C49 and C65 libraries (data not shown), with a very low abundance (4.17 and 0.12%, respectively). According to the GreenGenes database (DeSantis et al., 2006) used by QIIME for taxonomy and phylogeny sample C32 comprises also the proposed phylum *Parvarchaeota*. In the ARB tree only *Crenarchaeota* and *Euryarchaeota* phyla were observed, while *Parvarchaeota* appeared as a distinct lineage within *Euryarchaeota* (Supplementary Figure S1). *Parvarchaeota* is a recently proposed phylum (Rinke et al., 2013) that comprises of ultrasmall, uncultivated archaea, with a particular feature of aerobic respiration (Baker et al., 2010). So far, they have not been described in carbonate-impregnated microbial mats.

**Figure 4:**
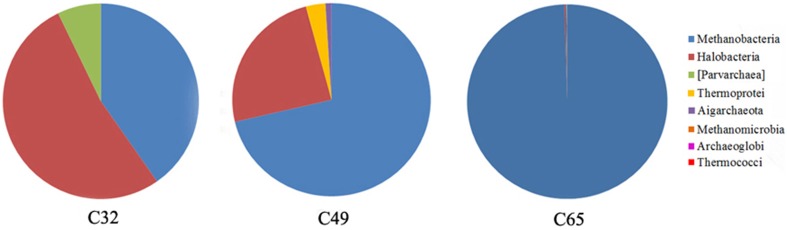
**Comparison of archaeal taxonomic diversity in the carbonate deposits from Ciocaia based on the percentage of sequencing reads attributed to Operational Taxonomic Units (OTUs)**. The C32 sample is dominated by halophiles, followed by methanogens and the proposed lineage [Parvarchaea]. As the temperatures increases, the dominance shifts toward methanogens (sample C49 and C65).

The *Crenarchaeota* 16S rRNA gene sequences from Ciocaia were assigned to two lineages: *Aigarchaeota* and *Thermoprotei* (Figure 4), being related to the Marine Benthic Group B from deep-sea sediments (Vetriani et al., 1999), the Hot Water Crenarchaeotic Group I (HWCGI) (Nunoura et al., 2010) (Supplementary Figure S1) and closely associated to members from the *Desulfurococaceae* family (Supplementary Figure S2). Members of the *Thermoprotei* class are known to be associated with high-temperature reservoirs (Tang et al., 2012; Lenchi et al., 2013).

*Euryarchaeota* 16S rRNA gene sequences were clustered into five classes: *Archaeoglobi, Halobacteria, Methanobacteria, Methanomicrobia*, and *Thermococci* (Figure 4; Supplementary Figures S1, S2). The 32°C sample is dominated by halophiles, followed by methanogens and the proposed lineage *Parvarchaea*. As the temperatures increases, the dominance in the investigated samples shifts toward methanogens (Figure 4).

Several halophilic Archaea genera were identified: *Halorhabdus, Halorubrum, Natronococcus, Natronomonas, Natronorubrum, Halorubellus*, and *Halovenus* (Supplementary Figures S1, S2). Even though they are obligate halophiles, they can survive in an environment with a much lower salinity than that of seawater (Purdy et al., 2003; Elshahed et al., 2004; Sayeh et al., 2010). Apparently, none of these genera were previously described in association with hot springs or hot spring carbonate deposits.

Identified methanogens belonged to the *Methanobacteria* and *Methanomicrobia* anaerobic groups that are usually associated with the deep biosphere and high temperature oil fields (Kimura et al., 2005; Ren et al., 2011; Tang et al., 2012; Lenchi et al., 2013). The high abundance of methanogens in the C65 carbonate sample may be linked to the low oxygen content of the heated fluid (Chapelle et al., 2002) or they could be brought up from the subsurface by the geothermal waterflow and immediately trapped between the carbonate crystals as they precipitate.

### Bacterial diversity and community structure

**Figure 5:**
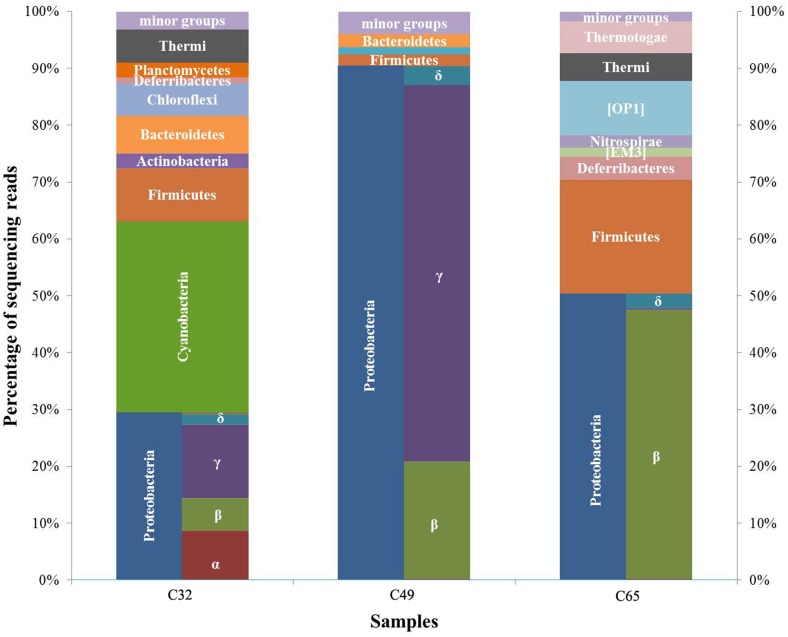
**Distribution of major bacterial phyla and classes in the carbonate deposits from Ciocaia based on the percent of sequencing reads that could be attributed to Operational Taxonomic Units (OTUs) within specific bacterial groups**.

Among the sequences representing the bacterial community, 66,367 were retrieved for C32, 123,284 for C49 and 63,255 for C65, respectively (Table 1). The number of observed OTUs decreased from 518 in C32 to 84 in C65. The ratio between the observed (Sobs) and estimated (Chao1) species richness ranged from 0.9 in C49 to 0.99 in C65 showing a good sequencing coverage. The taxonomy data cover a broad spectrum, the bacterial communities being composed of 39 different phyla (see Krona chart in the Supplementary Material). Twelve phyla and two candidate phyla had abundances higher than 1% (Figure 5) and are discussed below.

The dominant phylum in C32 was *Cyanobacteria* (~34%), followed by *Proteobacteria* (~29%), *Firmicutes* (~9%), *Bacteroidetes* (~7%), *Chloroflexi* (~6%), *Thermi* (~6%), *Actinobacteria* (~3%), *Planctomycetes* (~3%), and *Defferibacteres* (~1%). Within C49, there was a striking dominance of the phylum *Proteobacteria* (~91%), followed by *Firmicutes* and *Bacteroidetes* (each with 2%) and *Armantimonadetes* (~1%) as major groups. In C65, the dominant phyla were *Proteobacteria* (~50%), *Firmicutes* (~20%), [OP1] (~10%), *Defferibacteres, Thermi*, and *Thermotogae* (each with ~5%), [EM3] and *Nitrospirae* (each with less than 2%).

**Figure 6:**
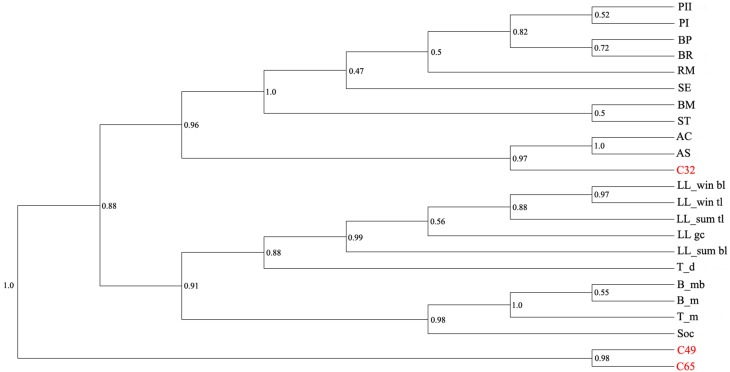
**Comparative analysis (UPGMA similarity tree) of microbial diversity in the Ciocaia samples with existing microbial mat/microbialite studies**. A closed-reference OTU picking was performed via QIIME (Caporaso et al., 2010a) at 97% sequence similarity, and the UPGMA tree constructed with unweighted-UniFrac (Lozupone and Knight, 2005). Sample codes: PI and PII, Pozas Azules I and II; BP, Pirate Channel; BR, Los Rapidos; RM, Rio Mesquites; SE, lagoon microbialite; BM, soft microbialite; ST, microbial mat; AC, columnar microbialite Alchichica lake; AS, spongy microbialite Alchichica lake (accession no. SRR350006; Centeno et al., 2012); LL_win bl, Salar de Llamara winter sample, bottom layer; LL_win tl, Salar de Llamara winter sample, top layer; LL_sum bl, Salar de Llamara summer sample, bottom layer; LL_sum tl, Salar de Llamara summer sample, top layer (accession no. SRR961678, SRR952918, SRR952917, SRR952915, SRR952913; Rasuk et al., 2014); T_d, Tebenquiche dome; B_mb, Brava microbialite; B_m, Brava mat; T_m, Tebenquiche mat (accession no. SRR627689, SRR627690, SRR627691, SRR627395; Farias et al., 2014); S, Socompa stromatolite (accession no. SRR329490; Farias et al., 2013).

There are noticeable differences in microbial communities between the three types of microenvironments (microbialites) (Figure 6). The C49 and C65 diversities are more similar to each other than to other microbial mats and microbialites, while the microbial diversity in C32 resembles to that of Alchichica crater lake (Figure 6; Centeno et al., 2012). This similarity can be a result of the fact that Cyanobacteria and Proteobacteria are both well represented in these mineral deposits. We extended the comparative analysis of diversity and taxonomic composition focusing primarily on phylum/class levels as different studies have used a variety of primers, sequencing and informatics approaches, which complicates quantitative and fine structure comparisons. The bacterial diversity of the mineralized mat collected at 32°C (sample C32) also presents a high degree of similarity with the intensively studied modern stromatolites from Highborne Cay, Bahamas (Myshrall et al., 2010; Khodadad and Foster, 2012), with other microbialites described in the alkaline Lake Alchichica, Mexico (Couradeau et al., 2011) or with mineral deposits from Ruidera Pools Natural Park, Spain (Santos et al., 2010). Cyanobacteria are well represented in these samples (12–57%), followed by Proteobacteria (18–30%). The main difference between these carbonate deposits is the dominance of *Gammaproteobacteria* in C32, rather than the *Alphaproteobacteria* documented in other modern stromatolite-like structures.

The bacterial diversity at 49°C and 65°C (samples C49 and C65, respectively) has unique characteristics when compared to other modern carbonate deposits worldwide. It is well known that *Proteobacteria* (mainly *Alphaproteobacteria*) tend to dominate carbonate deposits, accounting for 29–57% in carbonate deposits from several locations worldwide: Shark Bay, Australia (Papineau et al., 2005; Allen et al., 2009; Goh et al., 2009); Highborne Cay, Bahamas (Baumgartner et al., 2009; Mobberley et al., 2012); Lake Kinneret, Israel (Schwarz et al., 2007); freshwater environments from Mexico and Chile (Breitbart et al., 2009; Centeno et al., 2012; Farias et al., 2013, 2014); rhodoliths in Brazil (Cavalcanti et al., 2014). Foster and Green (2011) compared 16S rRNA gene datasets from modern carbonate deposits, developed in different environments, including hot springs, and observed that *Alphaproteobacteria* and *Cyanobacteria* were indeed the predominant groups in all the microbialites. The dominance of *Gammaproteobacteria* in C49 (75% of Proteobacteria) and *Betaproteobacteria* in C65 (90% of Proteobacteria) have not been previously observed in other carbonate deposits.

#### Carbonate-specific and carbonate-unspecific bacterial groups observed in the Ciocaia samples

Among the *Bacteria*, we identified several other taxa that have not been previously described in association with modern carbonates (Table 2).

**Table 2:** **Bacterial groups described for the first time in association with carbonate deposits**.

**OTU/Group**	**Taxonomic rank**	**Phylum/class**	**Sample**
4C0d-2/YS2	Class/Order	*Cyanobacteria* or [Melainabacteria]	C32
*Azoarcus*	Genus	*Betaproteobacteria*	C49
*Hydrogenophilus*	Genus	*Betaproteobacteria*	C49, C65
*Thermacetogenium*	Genus	*Betaproteobacteria*	C65
*Thermanaerobacterales*	Order	*Firmicutes*	C65
*Bacillus saliphilus* *Bacillus aurantiacus*	Species	*Firmicutes*	C32

At 32°C (sample C32), four cyanobacterial genera were identified (*Oscillatoria, Pseudoanabaena, Leptolyngbya*, and *Gloeobacter*), all common inhabitants of carbonate microbialites (Foster et al., 2009; Goh et al., 2009; Schulze-Makuch et al., 2013). Aside from them, we also identifed 17 sequences affiliated with the recently proposed phylum Melainabacteria (Di Rienzi et al., 2013). The taxonomy, phylogeny and physiological potentials of this Cyanobacteria-affiliated group of uncultured bacteria is not well understood (Di Rienzi et al., 2013; Soo et al., 2014). These deep branching organisms have been found in various environments, but have not been previously described in carbonate deposits associated with hot springs.

The most abundant order from *Alphaproteobacteria* at Ciocaia is represented by *Rhodobacteriales*, previously encountered in microbialites of Highborne Cay, Bahamas (Myshrall et al., 2010). The dominant *Betaproteobacteria* genera were *Azoarcus* and *Hydrogenophilus*. These two genera were previously found in several microbial communities, but not in carbonate impregnated microbialites. *Thiococcus*, was the dominant genus within *Gammaproteobacteria*. From *Deltaproteobacteria*, the dominant orders are *Myxococcales* and *Syntrophobacterales*, both being previously encountered in mineralized microbial mats (Foster and Green, 2011; Mobberley et al., 2013; Schneider et al., 2013; Ahrendt et al., 2014).

The 49°C sample (C49) was dominated by the phylum *Proteobacteria* (~91%), followed by *Firmicutes* and *Bacteroidetes* (each with 2%) and *Armantimonadetes* (~1%) as major groups. Distinctive about C49 is the fact that *Gamma*- and *Betaproteobacteria* account for about 75% of the whole bacterial partial 16S rRNA gene sequences. The dominant genera are *Idiomarina* (~34%) and *Halomonas* (~24%) from *Gammaproteobacteria*, together with *Hydrogenophilus* (16%) from *Betaproteobacteria*. The potential for carbonate precipitation by the halophilic members of the *Idiomarina* and *Halomonas* genera was previously documented (Heijs et al., 2006; González-Muñoz et al., 2008; Sánchez-Román et al., 2008).

The 65°C carbonate deposit (sample C65) was dominated by *Hydrogenophilus*, with 94% of the entire *Proteobacterial* sequences and about 47% of the entire library of partial 16S rRNA gene sequences. *Thermacetogenium* spp. is well represented, also, with about 17% of total. Organisms of this genus are very important for microbial mat development, by making a synthrophic association with hydrogenotrophic methanogens (Hattori et al., 2005). As far as we know, this taxon was never described as being a major group in other carbonate microbial mats.

The *Firmicutes* abundance was 9% in C32, 2% in C49 and 20% in C65. Nearly the entire population in C32 is composed of *Bacillus* sequences (*B. saliphilus* and *B. aurantiacus*), while in C49 the dominant order is *Clostridiales*, a group previously found in association with mineralizing microbial mats (Couradeau et al., 2011). In C65, the dominant group is *Thermoanaerobacterales* order that has never been described in carbonate microbialites.

### Functional diversity and possible mechanisms for genesis of carbonate crusts

The formation of carbonate deposits is quite common around continental hot springs, but an important question is whether that process is abiogenic, based solely on the saturation state of the water, or biogenic, where the mineral precipitation is influenced by the microbial consortium through their metabolism. According to Kim et al. (2012), the abiogenic precipitation prevails at the exit area of the geothermal water, while the level of biogenic influence on precipitation is in direct relationship with the increasing distance of the microbial mat from the geothermal water source and with the decrease of temperature. Based on that, in the sample closest to the exit hole of the drilling (C65), the mineral precipitation could be abiogenic. This possibility is supported by the SEM investigations (Figure 3F) that did not reveal a well-developed microbial mat in this sample. Also, the microbial diversity (methanogenic *Archaea, Hydrogenophilus* and *Thermacetogenium* genera, *Thermanaerobacterales* order) is that of a subsurface environment rather than of a terrestrial community (Kimura et al., 2005; Yamane et al., 2011; Tiago and Verissimo, 2012; Nyyssönen et al., 2014). The ascending hot water with high CO_2_ content crosses calcareous mudstone levels from where it dissolves and transports CaCO_3_ as Ca(HCO_3_)_2_, i.e., Ca^2+^ and HCO^−^_3_, together with archaeal and bacterial cells. At the surface, mainly due to the “low pressure effect,” calcium carbonate precipitates, trapping the cells. As we move farther apart from the exit point of the geothermal water, both the temperature and water flow decrease and the microbial mats start to thrive in those environments (Figures 1B,E, 2B,D). Thus, the carbonate mineralization could be influenced by both water and microbial mat (C49) or mainly by the microbial mat (C32), as the community is hydrated there only by sprinkling. CaCO_3_ precipitation by bacteria can be classified as biologically induced (Konhauser and Riding, 2012) because the cell has no control on the formation of minerals, these being the result of the interaction between the metabolism of microorganisms and the aquatic environment, resulting a so-called “alkalinity engine” (Dupraz et al., 2009) influencing the carbonate precipitation. The metabolic pathways that may be present in the microbial community at the three different temperatures were predicted based on the 16S rRNA gene data using the recently developed software PICRUSt (Langille et al., 2013). PICRUSt uses the OTU table of assigned taxa and their relative distribution to generate the relative abundance of functional categories based on sequenced genomes. Based on different KEGG functional gene ontology affiliation, the functions are arranged into three level subgroups (i.e., for level 1: metabolism, genetic information processing, environmental information processing, cellular processes, organismal systems, human diseases). To simplify analysis, only the “metabolism,” “genetic information processing,” “environmental information processing,” and “cellular processes” functions were analyzed further, as the categories of “organismal systems” and “human disease” were considered with limited relevance to environmental samples. Even though it is known that temperature plays a major role in shaping the diversity and function of microbial mats (Sharp et al., 2014), little variation in the predominant metabolic pathways was observed among the C32, C49, and C65 samples (Supplementary Figure S4). The single exceptions were the increase of carbon fixation through photosynthesis in C32 and that of methane-metabolizing pathways in C65 (data not shown). This can be explained by the high diversity of phototrophs and methanogenic archaea, respectively. The fact that no difference was observed via PICRUSt analysis (Supplementary Figure S4) could be attributed to a low quantity and quality of annotated genomes that are related to the species observed in the Ciocaia samples. Future updates are required as new genome sequences are sequenced and/or annotated.

**Figure 7:**
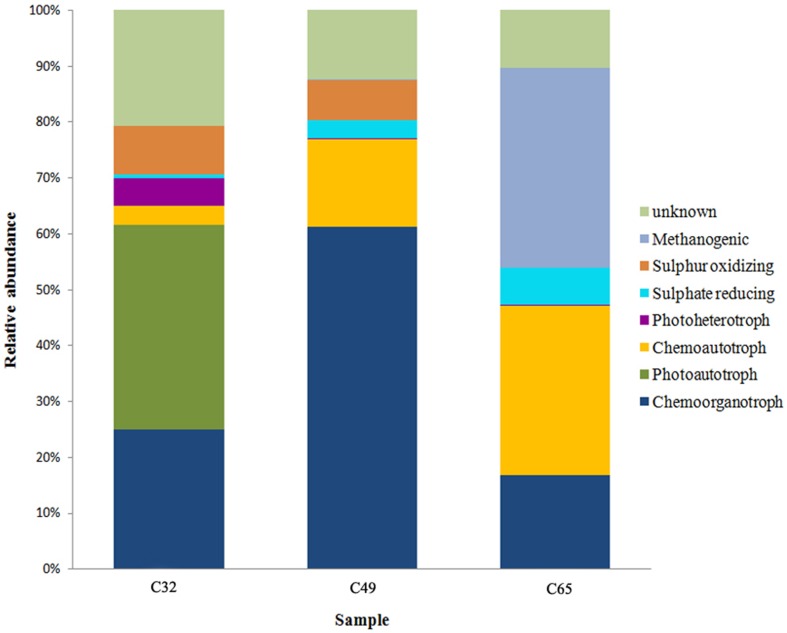
**Relative abundance distribution of putative bacterial functional groups in the carbonate deposits from Ciocaia**. The histograms are constructed based on the taxonomic affiliations inferred from the 16S rRNA genes.

We also analyzed dominant OTUs that were classified up to the species or genus level in an attempt to identify and predict specific metabolic groups and possible ecological interactions within each sample (Figure 7). However, there is no causality between species diversity and functional diversity, which requires caution when attributing functional roles within the microbial community based on rRNA classification. 16S rRNA-based identification is not always correlated with similarity of metabolic pathways and also some contributors may be hidden in OTUs that could not be assigned beyond the taxonomic level of phyla, class and order. In C49 the dominant genera *Idiomarina* and *Halomonas* comprise 60% of the entire 16S rRNA gene library. They are important promoters of carbonate precipitation through a unique high content of odd-iso-branched fatty acids in the cell membrane of *Idiomarina* species (González-Muñoz et al., 2008) or by creating an alkaline microenvironment around the cell by *Halomonas* species through oxidative deamination of amino acids (Heijs et al., 2006; Sánchez-Román et al., 2008). In HCO^−^_3_ rich mesothermal environments, the external pH can increase as inorganic carbon is consumed by phototrophic or chemolithoautotropic organisms faster than it can be replaced from the geothermal water (Hayashi et al., 1999; Badger et al., 2006; Raven, 2006). Phototrophs are dominant in C32 (Figure 7), being represented by both oxygenic and anoxygenic groups (*Cyanobacteria, Chloroflexi, Ectothiorhodospiraceae, Chromatiaceae*). The *Cyanobacteria-Chloroflexi* association has been previously documented in thermophilic microbial communities (Portillo et al., 2009) because in some cases, the oxygenic photosynthesis of cyanobacteria is dependent of the sulfide depletion by the anoxygenic *Chloroflexus* sp. (Jørgensen and Nelson, 1988; Kim, 1999). Sulphate-reducing bacteria (SRB), identified in all samples (Figure 7), can take part in increasing the environment's alkalinity by generating carbonate ions in the process of sulfate reduction. Part of the H_2_S consumed by anoxygenic photosynthesis may come from the activity of SRB as well. Heterotrophs, dominant in C49 and well represented in C32 and C65, can also increase the environment pH toward alkalinity by decomposing organic matter (Visscher et al., 2000; Konhauser, 2007; Baumgartner et al., 2009).

## Conclusions

To our knowledge, this is the first study of thermal carbonate deposits formed around an abandoned oil drill hole. It focused on sedimentary structures set in specific microenvironments at 32°C, 49°C, and 65°C around the geothermal spring from Ciocaia, Romania. The microbialites presented laminated macrostructures at lower temperatures and an irregular arrangement of carbonate crystals at 65°C. The microbial diversity in the three microenvironments either resembles that of other mineralized mats characterized in literature or presents a specific consortium of phyla and classes. Besides the archaeal and bacterial groups that are known to be associated with this kind of structures, there were several lineages observed for the first time in calcium carbonate deposits: the halophilic *Archaea* from the *Halobacteria* class, the non-photosynthetic, *Cyanobacteria*-related phylum *Melainabacteria*, several groups from *Proteobacteria* and *Firmicutes*. Future work is needed to determine the degree of biogenicity in each type of microbialite described in this paper. Morpho-structural studies, coupled with a metagenomic and metatranscriptomic approach, with stable isotope analysis and *in situ* functional studies and characterization of both surface and subsurface communities from Ciocaia will help determine the active components of the microbial mats and the degree of microbial contribution to the mineralization of calcium carbonate. Overall, the spring from Ciocaia emerges as a valuable site to probe microbes-minerals interrelationships along thermal and geochemical gradients.

### Conflict of interest statement

The authors declare that the research was conducted in the absence of any commercial or financial relationships that could be construed as a potential conflict of interest.

## Supplementary material

Supplementary Figure S1 **The phylogenetic placement of OTUs relative to known curated sequences deposited in the SILVA SSU Ref database (v115) using the ARB software package (Ludwig et al., 2004)**.

Supplementary Figure S2 **The phylogenetic placement of OTUs using the Maximum Likelihood algorithm in Mega 5 (Tamura et al., 2011) relative to manually selected 16S rRNA gene sequences from GenBank (NCBI database)**.

Supplementary Figure S3 **Rarefaction curves for the C32, C49 and C65 microbialite samples from Ciocaia**.

Supplementary Figure S4 **Relative abundance of gene counts in the C32, C49 and C65 microbialite samples from Ciocaia for selected functional KEGG pathways inferred from 16S rRNA gene data using PICRUSt (Langille et al., 2013)**.

## References

[B1] Ahrendt S. R. Mobberley J. M. Visscher P. T. Koss L. L. Foster J. S. (2014). Effects of elevated carbon dioxide and salinity on the microbial diversity in lithifying microbial mats. Minerals 4, 145–169 10.3390/min4010145

[B2] Allen M. A. Goh F. Burns B. P. Neilan B. A. (2009). Bacterial, archaeal and eukaryotic diversity of smooth and pustular microbial mat communities in the hypersaline lagoon of Shark Bay. Geology 7, 82–96 10.1111/j.1472-4669.2008.00187.x 19200148

[B3] Allwood A. C. Walter M. R. Kamber B. S. Marshall C. P. Burch I. W. (2006). Stromatolite reef from the Early Archaean era of Australia. Nature 441, 714–718. 10.1038/nature04764 16760969

[B4] Badger M. R. Price G. D. Long B. M. Woodger F. J. (2006). The environmental plasticity and ecological genomics of the cyanobacterial CO_2_ concentrating mechanism. J. Exp. Bot. 57, 249–265. 10.1093/jxb/eri286 16216846

[B5] Baker B. J. Comolli L. R. Dick G. J. Hauser L. J. Hyatt D. Dill B. D.. (2010). Enigmatic, ultrasmall, uncultivated archaea. Proc. Natl. Acad. Sci. U.S.A. 107, 8806–8811. 10.1073/pnas.0914470107 20421484 PMC2889320

[B6] Baumgartner L. K. Dupraz C. Buckley D. H. Spear J. R. Pace N. R. Visscher P. T. (2009). Microbial species richness and metabolic activities in hypersaline microbial mats: insight into biosignature formation through lithification. Astrobiology 9, 861–874. 10.1089/ast.2008.0329 19968463

[B7] Bendea C. Bendea G. Rosca M. Cucueteanu D. (2013). Current status of geothermal energy production and utilization in Romania. J. Sustain Energ. 4, 1–9.

[B8] Berelson W. M. Corsetti F. A. Pepe-Ranney C. Hammond D. E. Beaumont W. Spear J. R. (2011). Hot spring siliceous stromatolites from Yellowstone National Park: assessing growth rate and laminae formation. Geobiology 9, 411–424. 10.1111/j.1472-4669.2011.00288.x 21777367

[B9] Braissant O. Decho A. W. Dupraz C. Glunk C. Przekop K. M. Visscher P. T. (2007). Exopolymeric substances of sulfate-reducing bacteria: interactions with calcium at alkaline pH and implication for formation of carbonate minerals. Geobiology 5, 401–411 10.1111/j.1472-4669.2007.00117.x

[B10] Brasier M. McLoughlin N. Green O. Wacey D. (2006). A fresh look at the fossil evidence for early Archaean cellular life. Philos. Trans. R. Soc. B 361, 887–902 10.1098/rstb.2006.1835 PMC1578727 16754605

[B11] Breitbart M. Hoare A. Nitti A. Siefert, J, Haynes M. Dinsdale E.. (2009). Metagenomic and stable isotopic analyses of modern freshwater microbialites in Cuatro Ciénegas, Mexico. Environ. Microbiol. 11, 16–34. 10.1111/j.1462-2920.2008.01725.x 18764874

[B12] Caporaso J. G. Bittinger K. Bushman F. D. DeSantis T. Z. Andersen G. L. Knight R. (2010b). PyNAST: a flexible tool for aligning sequences to a template alignment. Bioinformatics 26, 266–267. 10.1093/bioinformatics/btp636 19914921 PMC2804299

[B13] Caporaso J. G. Kuczynski J. Stombaugh J. Bittinger K. Bushman F. D. Costello E. K.. (2010a). QIIME allows analysis of high-throughput community sequencing data. Nat. Methods 7, 335–336. 10.1038/nmeth.f.303 20383131 PMC3156573

[B14] Cavalcanti G. S. Gregoracci G. B. dos Santos E. O. Silveir C. B. Meirelles P. M. Longo L.. (2014). Physiologic and metagenomic attributes of the rhodoliths forming the largest CaCO_3_ bed in the South Atlantic Ocean. ISME J. 8, 52–62. 10.1038/ismej.2013.133 23985749 PMC3869012

[B15] Centeno C. M. Legendre P. Beltran Y. Alcantara-Hernandez R. J. Lidstrom U. E. Ashby M. N.. (2012). Microbialite genetic diversity and composition related to environmental variables. FEMS Microbiol. Ecol. 82, 724–735. 10.1111/j.1574-6941.2012.01447.x 22775797

[B16] Chapelle F. H. O'Neill K. Bradley P. M. Methe B. A. Ciufo S. A. Knobel L. L.. (2002). A hydrogen-based subsurface microbial community dominated by methanogens. Nature 415, 312–315. 10.1038/415312a 11797006

[B17] Coman C. Bica A. Drugã B. Barbu-Tudoran L. Dragoş N. (2011). Methodological constraints in the molecular biodiversity study of a thermomineral spring cyanobacterial mat: a case study. Anton. Leeuw. Int. J. G 92, 271–281. 10.1007/s10482-010-9486-5 20665239

[B18] Coman C. Bica A. Drugã B. Barbu-Tudoran L. Dragoş N. (2012). A microbial mat developed around a man-made geothermal spring from Romania: structure and cyanobacterial composition, in Microbial Mats in Siliciclastic Depositional Systems through Time, SEPM Special Publication, Vol. 101, eds Noffke N. Chafetz H. (Tulsa, OK: SEPM Society for Sedimentary Geology), 47–53.

[B19] Coman C. Drugã B. Hegedus A. Sicora C. Dragoş N. (2013). Archaeal and bacterial diversity in two hot spring microbial mats from a geothermal region in Romania. Extremophiles 17, 523–534. 10.1007/s00792-013-0537-5 23568449

[B20] Couradeau E. Benzerara K. Moreira D. Gérard E. Kaźmierczak, J, Tavera T.. (2011). Prokaryotic and eukaryotic community structure in field and cultured microbialites from the Alkaline Lake Alchichica (Mexico). PLoS ONE 6:e28767. 10.1371/journal.pone.0028767 22194908 PMC3237500

[B21] DeSantis T. Z. Hugenholtz P. Larsen N. Rojas M. Brodie E. L. Keller K.. (2006). Greengenes, a chimera-checked 16S rRNA gene database and workbench compatible with ARB. Appl. Environ. Microbiol. 72, 5069–5072. 10.1128/AEM.03006-05 16820507 PMC1489311

[B22] Di Rienzi S. C. Sharon I. Wrighton K. C. Koren O. Hug L. A. Thomas B. C.. (2013). The human gut and groundwater harbor non-photosynthetic bacteria belonging to a new candidate phylum sibling to Cyanobacteria. eLife 2:e01102. 10.7554/eLife.01102 24137540 PMC3787301

[B23] Dupraz C. Reid R. P. Braissant O. Decho A. W. Norman R. S. Visscher P. T. (2009). Processes of carbonate precipitation in modern microbial mats. Earth Sci. R. 96, 141–162 10.1016/j.earscirev.2008.10.005

[B24] Dupraz C. Visscher P. T. (2005). Microbial lithification in marine stromatolites and hypersaline mats. Trends Microbiol. 13, 429–438. 10.1016/j.tim.2005.07.008 16087339

[B25] Edgar R. (2013). UPARSE: highly accurate OTU sequences from microbial amplicon reads. Nat. Methods 10, 996–998. 10.1038/nmeth.2604 23955772

[B26] Elshahed M. S. Najar F. Z. Roe B. A. Oren A. Dewers T. A. Krumholz L. R. (2004). Survey of archaeal diversity reveals an abundance of halophilic Archaea in a low-salt, sulfide- and sulfur-rich spring. Appl. Environ. Microbiol. 70, 2230–2239. 10.1128/AEM.70.4.2230-2239.2004 15066817 PMC383155

[B27] Farias M. E. Contreras M. Rasuk M. C. Kurth D. Flores M. R. Poire D. G.. (2014). Characterization of bacterial diversity associated with microbial mats, gypsum evaporites and carbonate microbialites in thalassic wetlands: Tebenquiche and La Brava, Salar de Atacama, Chile. Extremophiles 18, 311–329. 10.1007/s00792-013-0617-6 24442191

[B28] Farias M. E. Rascovan N. Toneatti D. M. Albarracín V. H. Flores M. R. Poiré D. G.. (2013). The discovery of stromatolites developing at 3570 m above sea level in a high-altitude volcanic lake Socompa, Argentinean Andes. PLoS ONE 8:e53497. 10.1371/journal.pone.0053497 23308236 PMC3538587

[B29] Fierer N. Leff J. W. Adams B. J. Nielsen U. N. Bates S. T.. (2012). Cross-biome metagenomics analyses of soil microbial communities and their functional attributes. Proc. Natl. Acad. Sci. U.S.A. 109, 21390–21395. 10.1073/pnas.1215210110 23236140 PMC3535587

[B30] Foster J. S. Green S. J. (2011). Microbial diversity in modern stromatolites Stromatolites: interaction of microbes with sediments. Cell Origin Life Ext. 18, 383–405 10.1007/978-94-007-0397-1_17

[B31] Foster J. S. Green S. J. Ahrendt S. R. Golubic S. Reid R. P. Hetherington K. L.. (2009). Molecular and morphological characterization of cyanobacterial diversity in the stromatolites of Highborne Cay, Bahamas. ISME J. 3, 573–587. 10.1038/ismej.2008.129 19148145

[B32] Furnes H. Banerjee N. R. Muehlenbachs K. Staudigel H. de Wit M. (2004). Early life recorded in Archean pillow lavas. Science 304, 578–581. 10.1126/science.1095858 15105498

[B33] Goh F. Allen M. A. Leuko S. Kawaguchi T. Decho A. W. Burns B. P.. (2009). Determining the specific microbial populations and their spatial distribution within the stromatolite ecosystem of Shark Bay ISME J. 3, 383–396. 10.1038/ismej.2008.114 19092864

[B34] González-Muñoz M. T. De Linares C. Martínez-Ruiz F. Morcillo F. Martín-Ramos D. Arias J. M. (2008). Ca-Mg kutnahorite and struvite production by *Idiomarina* strains at modern seawater salinities. Chemosphere 72, 465–472. 10.1016/j.chemosphere.2008.02.010 18355891

[B36] Guindon S. Gascuel O. (2003). A simple, fast and accurate method to estimate large phylogenies by maximum-likelihood. Syst. Biol. 52, 696–704. 10.1080/10635150390235520 14530136

[B37] Hattori S. Galushko A. S. Kamagata Y. Schink B. (2005). Operation of the CO dehydrogenase/acetyl coenzyme A pathway in both acetate oxidation and acetate formation by the syntrophically acetate-oxidizing bacterium *Thermoacetogenium phaeum*. J. Bacteriol. 187, 3471–3476. 10.1128/JB.187.10.3471-3476.2005 15866934 PMC1111993

[B38] Hayashi N. R. Ishida T. Yokota A. Kodama T. Igarashi Y. (1999). *Hydrogenophilus thermoluteolus* gen nov, sp nov, a thermophilic, facultatively chemolithoautotrophic, hydrogen-oxidizing bacterium. Int. J. Syst. Bacteriol. 49, 783–786. 10.1099/00207713-49-2-783 10319503

[B39] Heijs S. K. Aloisi G. Bouloubassi I. Pancost R. D. Pierre C. Sinninghe-Damsté J. S. (2006). Microbial community structure in three deep-sea carbonate crusts. Microbial. Ecol. 52, 451–462 10.1007/s00248-006-9099-8 16909345

[B40] Hofmann H. J. Grey K. Hickman A. H. Thorpe R. I. (1999). Origin of 345 Ga Coniform stromatolites in the Warrawoona Group, Western Australia. Bull. Geol. Soc. Am. 111, 1256–1262.

[B41] Huson D. H. Scornavacca C. (2012). Dendroscope 3: an interactive tool for rooted phylogenetic trees and networks. Syst. Biol. 61, 1061–1067. 10.1093/sysbio/sys062 22780991

[B42] Jørgensen B. B. Nelson C. N. (1988). Bacterial zonation, photosynthesis, and spectral light distribution in hot spring mirobial mats of Iceland. Microbial. Ecol. 16, 133–147. 10.1007/BF02018909 24201567

[B43] Kawaguchi T. Decho A. W. (2002a). A laboratory investigation of cyanobacterial extracellular polymeric secretions (EPS) in influencing CaCO_3_ polymorphism. J. Cryst. Growth 240, 230–235 10.1016/S0022-0248(02)00918-1

[B44] Kawaguchi T. Decho A. W. (2002b). Characterization of extracellular polymeric secretions (EPS) from modern soft marine stromatolites (Bahamas) and its inhibitory effect on CaCO_3_ precipitation. Prep. Biochem. Biotechnol. 32, 51–63. 10.1081/PB-120013161 11934077

[B45] Khodadad C. L. M. Foster J. S. (2012). Metagenomic and metabolic profiling of nonlithifying and lithifying stromatolitic mats of Highborne Cay, The Bahamas. PLoS ONE 7:e38229. 10.1371/journal.pone.0038229 22662280 PMC3360630

[B46] Kim B. H. (1999). Ecology of a cyanobacterial mat community in a Korean thermal wastewater stream. Aquat. Ecol. 33, 331–338 10.1023/A:1009986606414

[B47] Kim J. W. Kogure T. Yang K. Kim S. T. Jang Y. N. Baik H. S. (2012). The characterization of CaCO_3_ in a geothermal environment: A SEM/TEM-EELS study. Clay Clay Miner. 60, 484–495 10.1346/CCMN.2012.0600505

[B48] Kimura H. Sugihara M. Yamamoto H. Patel B. K. C. Kato K. Hanada S. (2005). Microbial community in a geothermal aquifer associated with the subsurface of the great Artesian Basin, Australia. Extremophiles 9, 407–414. 10.1007/s00792-005-0454-3 15980939

[B49] Konhauser K. (2007). Introduction of Geomicrobiology. Oxford: Blackwell Publishing Ltd 425.

[B50] Konhauser K. Riding R. (2012). Bacterial biomineralization, in Fundamentals of Geobiology, eds Knoll A. Canfield D. Konhauser K. (Chichester: Wiley-Blackwell), 105–130.

[B51] Langille M. G. Zaneveld J. Caporaso J. G. McDonald D. Knights D. Reyes J. A.. (2013). Predictive functional profiling of microbial communities using 16S rRNA marker gene sequences. Nat. Biotechnol. 31, 814–821. 10.1038/nbt.2676 23975157 PMC3819121

[B52] Lenchi N. Ýnceoğlu Ö. Kebbouche-Gana S. Gana M. L. Llirós M. Servais P.. (2013). Diversity of microbial communities in production and injection waters of algerian oilfields revealed by 16S rRNA Gene Amplicon 454 Pyrosequencing. PLoS ONE 8:e66588. 10.1371/journal.pone.0066588 23805243 PMC3689743

[B53] Liu Z. Lozupone C. Hamady M. Bushman F. D. Knight R. (2007). Short pyrosequencing reads suffice for accurate microbial community analysis. Nucleic Acids Res. 35, e120. 10.1093/nar/gkm541 17881377 PMC2094085

[B106] Lozupone C. Knight R. (2005). UniFrac: a new phylogenetic method for comparing microbial communities. Appl. Environ. Microbiol. 71, 8228–8235. 10.1128/AEM.71.12.8228-8235.2005 16332807 PMC1317376

[B54] Ludwig W. Strunk O. Westram R. Richter L. Meier H. Yadhukumar. (2004). ARB: a software environment for sequence data. Nucleic Acids Res. 32, 1363–1371. 10.1093/nar/gkh293 14985472 PMC390282

[B55] Lundberg D. S. Yourstone S. Mieczkowski P. Jones C. D. Dangl J. L. (2013). Practical innovations for high-throughput amplicon sequencing. Nat. Methods 10, 999–1002. 10.1038/nmeth.2634 23995388

[B56] Martin M. (2011). Cutadapt removes adapter sequences from high-throughput sequencing reads. EMBnet J. 17, 10–12 10.14806/ej.17.1.200

[B57] McDonald D. Price M. N. Goodrich J. Nawrocki E. P. DeSantis T. Z. Probst A.. (2012). An improved Greengenes taxonomy with explicit ranks for ecological and evolutionary analyses of bacteria and archaea. ISME J. 6, 610–618. 10.1038/ismej.2011.139 22134646 PMC3280142

[B58] McLoughlin N. Wilson L. Brasier M. D. (2008). Growth of synthetic stromatolites and wrinkle structures in the absence of microbes—implications for the early fossil record. Geobiology 6, 95–105. 10.1111/j.1472-4669.2007.00141.x 18380872

[B59] Mobberley J. M. Khodadad K. L. M. Foster J. S. (2013). Metabolic potential of lithifying cyanobacteria-dominated thrombolitic mats. Photosynth. Res. 118, 125–140. 10.1007/s11120-013-9890-6 23868401 PMC5766932

[B60] Mobberley J. M. Ortega M. C. Foster J. S. (2012). Comparative microbial diversity analyses of modern marine thrombolitic mats by barcoded pyrosequencing. Environ. Microbiol. 14, 82–100. 10.1111/j.1462-2920.2011.02509.x 21658172

[B61] Moore D. D. (1995). Preparation and analysis of DNA, in Short Protocols in Molecular Biology, 3rd Edn, eds Ausubel F. Brent R. Kingston R. Moore D. Seidman J. G. Smith J. (New York, NY: John Wiley & Sons), 2–3.

[B62] Myshrall K. L. Mobberley J. M. Green S. J. Visscher P. T. Havemann S. A. Reid R. P.. (2010). Biogeochemical cycling and microbial diversity in the thrombolitic microbialites of Highborne Cay, Bahamas. Geobiology 8, 337–354. 10.1111/j.1472-4669.2010.00245.x 20491947

[B63] Nitti A. Daniels C. A. Siefert J. Souza V. Hollander D. Breitbart M. (2012). Spatially resolved genomic, stable isotopic, and lipid analyses of a modern freshwater microbialite from Cuatro Ciénegas, Mexico. Astrobiology 12, 685–698. 10.1089/ast.2011.0812 22882001 PMC3426887

[B64] Noffke N. Beukes N. Bower D. Hazen R. M. Swift D. J. P. (2008). An actualistic perspective into Archean worlds-(cyano-)bacterially induced sedimentary structures in the siliciclastic Nhlazatse Section, 2.9 Ga Pongola Supergroup, South Africa. Geobiology 6, 5–20. 10.1111/j.1472-4669.2007.00118.x 18380882

[B65] Nunoura T. Takaki Y. Kakuta J. Nishi S. Sugahara J. Kazama H.. (2010). Insights into the evolution of Archaea and eukaryotic protein modifier systems revealed by the genome of a novel archaeal group. Nucleic Acids Res. 39, 3204–3223. 10.1093/nar/gkq1228 21169198 PMC3082918

[B66] Nyyssönen M. Hultman J. Ahonen L. Kukkonen I. Paulin L. Laine P.. (2014). Taxonomically and functionally diverse microbial communities in deep crystalline rocks of the fennoscandian shield. ISME J. 8, 126–138. 10.1038/ismej.2013.125 23949662 PMC3869007

[B67] Obst M. Dynes J. J. Lawrence J. R. Swerhone G. D. W. Benterara K. Kaznatcheev K. (2009). Precipitation of amorphous CaCO_3_ (aragonite-like) by cyanobacteria: a STXM study of the influence of EPS on the nucleation process. Geochim. Cosmochim. Ac. 73, 4180–4198 10.1016/j.gca.2009.04.013

[B68] Ondov B. D. Bergman N. H. Phillippy A. M. (2011). Interactive metagenomic visualization in a Web browser. BMC Bioinformatics 12:385. 10.1186/1471-2105-12-385 21961884 PMC3190407

[B69] Papineau D. Walker J. J. Mojzsis S. J. Pace N. R. (2005). Composition and structure of microbial communities from stromatolites of Hamelin Pool in Shark Bay, Western Australia. Appl. Environ. Microbiol. 71, 4822–4832. 10.1128/AEM.71.8.4822-4832.2005 16085880 PMC1183352

[B70] Pepe-Ranney C. Berelson W. M. Corsetti F. A. Treants M. Spear J. R. (2012). Cyanobacterial construction of hot spring siliceous stromatolites in Yellowstone National Park. Environ. Microbiol. 14, 1182–1197. 10.1111/j.1462-2920.2012.02698.x 22356555

[B71] Portillo M. C. Sririn V. Kanoksilapatham W. Gonzalez J. M. (2009). Differential microbial communities in hot spring mats from Western Thailand. Extremophiles 13, 321–331. 10.1007/s00792-008-0219-x 19109691

[B72] Posada D. (2003). jModelTest: phylogenetic model averaging. Mol. Evol. Biol. 25, 1253–1256. 10.1093/molbev/msn083 18397919

[B73] Price M. N. Dehal P. S. Arkin A. P. (2010). FastTree 2—Approximately maximum-likelihood trees for large alignments. PLoS ONE 5:e9490. 10.1371/journal.pone.0009490 20224823 PMC2835736

[B74] Pruesse E. Peplies J. Glöckner F. O. (2012). SINA: accurate high-throughput multiple sequence alignment of ribosomal RNA genes. Bioinformatics 28, 1823–1829. 10.1093/bioinformatics/bts252 22556368 PMC3389763

[B75] Purdy K. J. Nedwell D. B. Embley T. M. (2003). Analysis of the sulfate-reducing bacterial and methanogenic archaeal populations in contrasting Antarctic sediments. Appl. Environ. Microbiol. 69, 3181–3191. 10.1128/AEM.69.6.3181-3191.2003 12788715 PMC161550

[B76] Rasuk C. R. Kurth D. Flores M. R. Contreras M. Novoa F. Poire D.. (2014). Microbial characterization of microbial ecosystems associated to evaporites domes of gypsum in Salar de Llamara in Atacama desert. Microb. Ecol. 68, 483–494. 10.1007/s00248-014-0431-4 24859438

[B77] Raven J. A. (2006). Sensing inorganic carbon: CO_2_ and HCO^−^_3_. Biochem. J. 396, e5–e7. 10.1042/BJ20060574 16703664 PMC1462711

[B78] Reid R. P. Visscher P. T. Decho A. W. Stolz J. F. Bebout B. M. Dupraz C.. (2000). The role of microbes in accretion, lamination and early lithification of modern marine stromatolites. Nature 406, 989–992. 10.1038/35023158 10984051

[B79] Ren H. Y. Zhang X. J. Song Z. Y. Rupert W. Gao G. J. Guo S. X.. (2011). Comparison of microbial community compositions of injection and production well samples in a long-term water-flooded petroleum reservoir. PLoS ONE 6:e23258. 10.1371/journal.pone.0023258 21858049 PMC3156122

[B80] Reyes-Escogido L. Balam-Chi M. Rodríguez-Buenfil I. Valdés J. Kameyama L. Martínez-Pérez F. (2010). Purification of bacterial genomic DNA in less than 20 min using chelex-100 microwave: examples from strains of lactic acid bacteria isolated from soil samples. Antonie Van Leeuwenhoek 98, 465–474. 10.1007/s10482-010-9462-0 20556655

[B81] Rinke C. Schwientek P. Sczyrba A. Ivanova N. N. Anderson I. J. Cheng J. F.. (2013). Insights into the phylogeny and coding potential of microbial dark matter. Nature 499, 431–437. 10.1038/nature12352 23851394

[B82] Sánchez-Román M. Vasconcelos C. Schmid T. Dittrich M. McKenzie J. A. Zenobi R. (2008). Aerobic microbial dolomite at the nanometer scale: implications for the geologic record Geology 36, 879–882 10.1130/G25013A.1

[B83] Santos F. Pena A. Nogales B. Soria-Soria E. García del Cura M. A. González-Martín J. A.. (2010). Bacterial diversity in dry modern freshwater stromatolites from Ruidera Pools Natural Park, Spain. Syst. Appl. Microbiol. 33, 209–221. 10.1016/j.syapm.2010.02.006 20409657

[B84] Sayeh R. Birrien J. L. Alain K. Barbier G. Hamdi M. Prieur D. (2010). Microbial diversity in Tunisian geothermal springs as detected by molecular and culture-based approaches Extremophiles 14, 501–514. 10.1007/s00792-010-0327-2 20835839

[B85] Schneider D. Arp G. Reimer A. Reitner J. Daniel R. (2013). Phylogenetic analysis of a microbialite - forming microbial mat from a hypersaline lake of the Kiritimati Atoll, Central Pacific. PLoS ONE 8:e66662. 10.1371/journal.pone.0066662 23762495 PMC3677903

[B86] Schopf J. W. (2002). The fossil record: tracing the roots of the cyanobacterial lineage, in The Ecology of Cyanobacteria. Their Diversity in Time and Space, eds Whitton B. A. Potts M. (Dordrecht: Kluwer Academic Press), 1–17.

[B87] Schopf J. W. (2006). The first billion years: when did life emerge? Elements 2, 229–233 10.2113/gselements.2.4.229

[B88] Schulze-Makuch D. Lim D. S. S. Laval B. Turse C. Antonio M. R. S. Chan O.. (2013). Pavilion Lake microbialites: morphological, molecular, and biochemical evidence for a cold-water transition to colonial aggregates. Life 3, 21–37. 10.3390/life3010021 25371330 PMC4187197

[B89] Schwarz J. I. K. Eckert W. Conrad R. (2007). Community structure of Archaea and Bacteria in a profundal lake sediment, Lake Kinneret (Israel). Syst. Appl. Microbiol. 30, 239–254. 10.1016/j.syapm.2006.05.004 16857336

[B90] Sharp C. E. Brady A. L. Sharp G. H. Grasby S. E. Stott M. B. Dunfield P. F. (2014). Humboldt's spa: microbial diversity is controlled by temperature in geothermal environments. ISME J. 8, 1166–1174. 10.1038/ismej.2013.237 24430481 PMC4030231

[B91] Soo R. M. Skennerton C. T. Sekiguchi Y. Imelfort M. Paech S. J. Dennis P. G. (2014). Photosynthesis is not a universal feature of the phylum Cyanobacteria. PeerJ. PrePrints 2:e204ve1 10.7287/peerj.preprints.204v2

[B92] Stoddard S. V. Smith B. J. Hein R. Roller B. R. K. Schmidt T. M. (2014). rrnDB: improved tools for interpreting rRNA gene abundance in bacteria and archaea and a new foundation for future development. Nucl. Acid. Res. 43, D593–D598. 10.1093/nar/gku1201 25414355 PMC4383981

[B93] Tamura K. Peterson D. Peterson N. Stecher G. Nei M. Kumar S. (2011). MEGA5: molecular evolutionary genetics analysis using maximum likelihood, evolutionary distance, and maximum parsimony. Methods Mol. Biol. Evol. 28, 2731–2739. 10.1093/molbev/msr121 21546353 PMC3203626

[B94] Tang Y. Q. Li Y. Zhao J. Y. Chi C. Q. Huang L. X. Dong H. P.. (2012). Microbial communities in long-term, water-flooded petroleum reservoirs with different *in situ* temperatures in the Huabei Oilfield, China. PLoS ONE 7:e33535. 10.1371/journal.pone.0033535 22432032 PMC3303836

[B95] Ţenu A. Constantinescu T. Davidescu F. Nuti S. Noto P. Squarci P. (1981). Research on the thermal waters of the Western Plain of Romania. Geothermics 10, 1–28. 10.1016/0375-6505(81)90021-323568449

[B96] Tiago I. Verissimo A. (2012). Microbial and functional diversity of a subterrestrial high pH groundwater associated to serpentinization. Environ. Microbiol. 5, 1687–1706 10.1111/1462-2920.12034 23731249

[B97] Tice M. M. Lowe D. R. (2004). Photosynthetic microbial mats in the 3,416-Myr-old ocean. Nature 431, 549–552. 10.1038/nature02888 15457255

[B98] Vetriani C. Jannasch H. W. MacGregor B. J. Stahl D. A. Reysenbach A. L. (1999). Population structure and phylogenetic characterization of marine benthic Archaea in deep-sea sediments. Appl. Environ. Microbiol. 65, 4375–4384. 10508063 10.1128/aem.65.10.4375-4384.1999 PMC91581

[B99] Visscher P. T. Reid R. P. Bebout B. M. (2000). Microscale observations of sulfate reduction: correlation of microbial activity with lithified micritic laminae in modern marine stromatolites. Geology 28, 919–922 10.1130/0091-7613(2000)28<919:MOOSRC>2.0.CO;2

[B100] Walsh M. M. (1992). Microfossils and possible microfossils from the early archean onverwacht group, barberton mountain land, South Africa. Precambrian Res. 54, 271–292. 10.1016/0301-9268(92)90074-X 11540926

[B101] Walter M. R. Buick R. Dunlop J. S. R. (1980). Stromatolites, 3,400–3,500 Myr old from the North Pole area, Western Australia. Nature 284, 443–445 10.1038/284443a0

[B102] Wang Q. Garrity G. M. Tiedje J. M. Cole J. R. (2007). Naive Bayesian classifier for rapid assignment of rRNA sequences into the new bacterial taxonomy. Appl. Environ. Microbiol. 73, 5261–5267. 10.1128/AEM.00062-07 17586664 PMC1950982

[B103] Werner J. J. Koren O. Hugenholtz P. DeSantis T. Z. Walters W. A. Caporaso J. G.. (2012). Impact of training sets on classification of high-throughput bacterial 16s rRNA gene surveys. ISME J. 6, 94–103. 10.1038/ismej.2011.82 21716311 PMC3217155

[B104] Westall F. de Vries S. T. Nijman W. Rouchon V. Orberger B. Pearson (2006). The 3446 Ga “Kitty's Gap Chert,” an early Archean microbial ecosystem. GSA Special Paper 405, 105–131 10.1130/2006.2405(07)

[B105] Yamane K. Hattori Y. Ohtagaki H. Fujiwara K. (2011). Microbial diversity with dominance of 16S rRNA gene sequences with high GC contents at 74 and 98°C subsurface crude oil deposits in Japan. FEMS Microbiol. Ecol. 76, 220–235. 10.1111/j.1574-6941.2011.01044.x 21223337

