# Supplementary figures and images for: Structure, mineralogy, and microbial diversity of geothermal spring microbialites associated with a deep oil drilling in Romania
Source: Front Microbiol. 2015 Mar 30;6:253. doi: 10.3389/fmicb.2015.00253 (PMC4378309; doi:10.3389/fmicb.2015.00253)

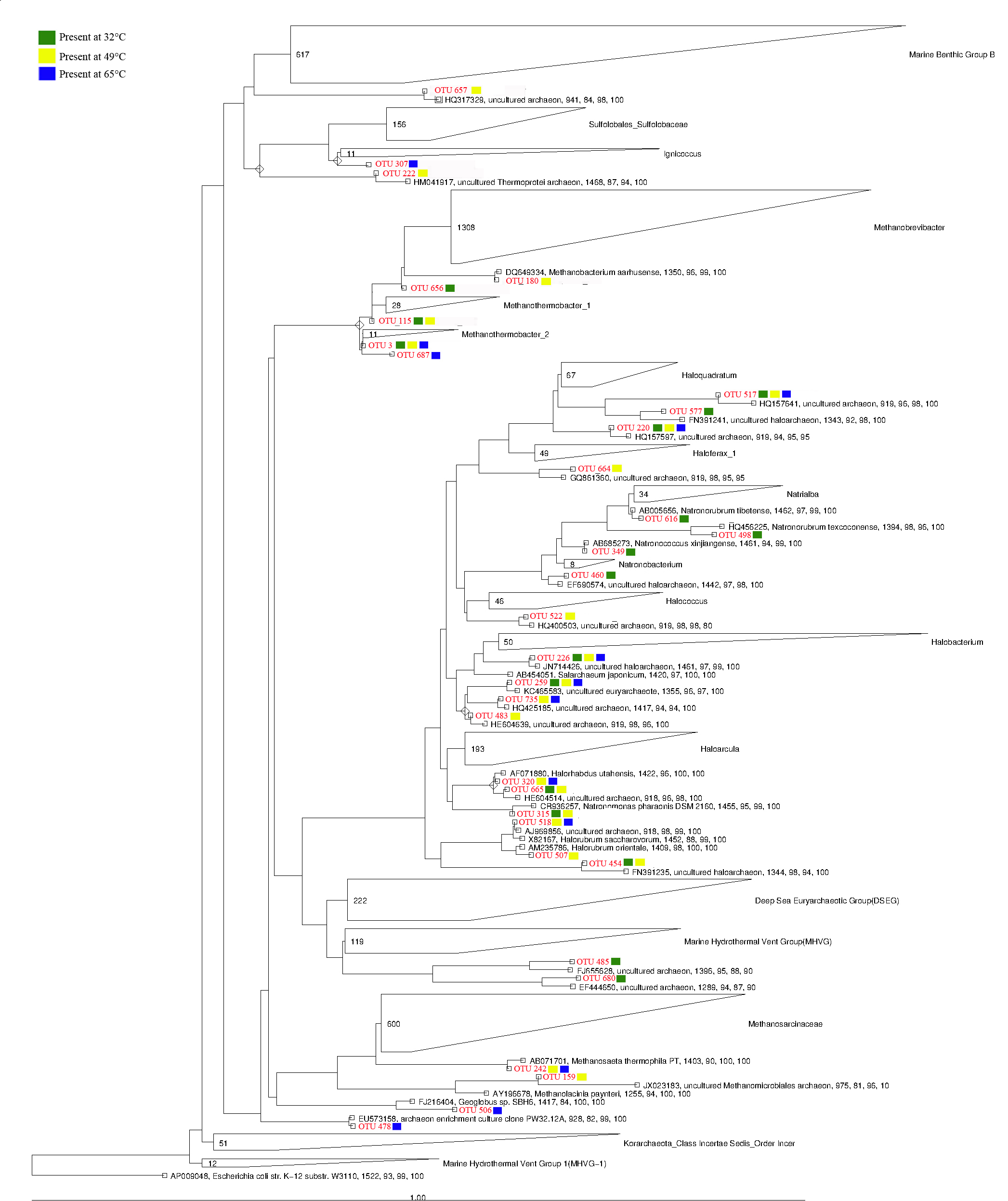

Supplement: Supplementary Figure S1 — The phylogenetic placement of OTUs relative to known curated sequences deposited in the SILVA SSU Ref database (v115) using the ARB software package (Ludwig et al., 2004). [file Image1.JPEG]

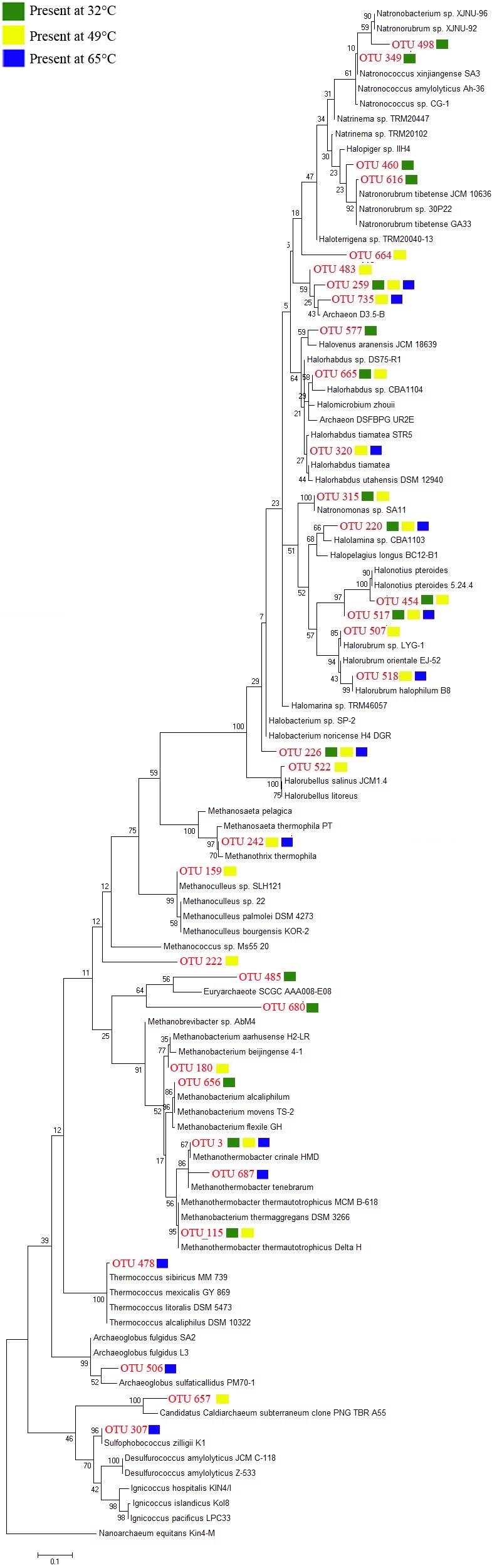

Supplement: Supplementary Figure S2 — The phylogenetic placement of OTUs using the Maximum Likelihood algorithm in Mega 5 (Tamura et al., 2011) relative to manually selected 16S rRNA gene sequences from GenBank (NCBI database). [file Image2.JPEG]

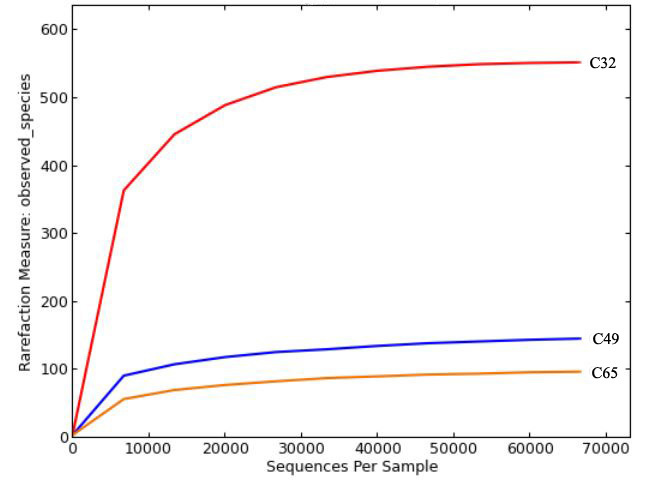

Supplement: Supplementary Figure S3 — Rarefaction curves for the C32, C49 and C65 microbialite samples from Ciocaia. [file Image3.JPEG]

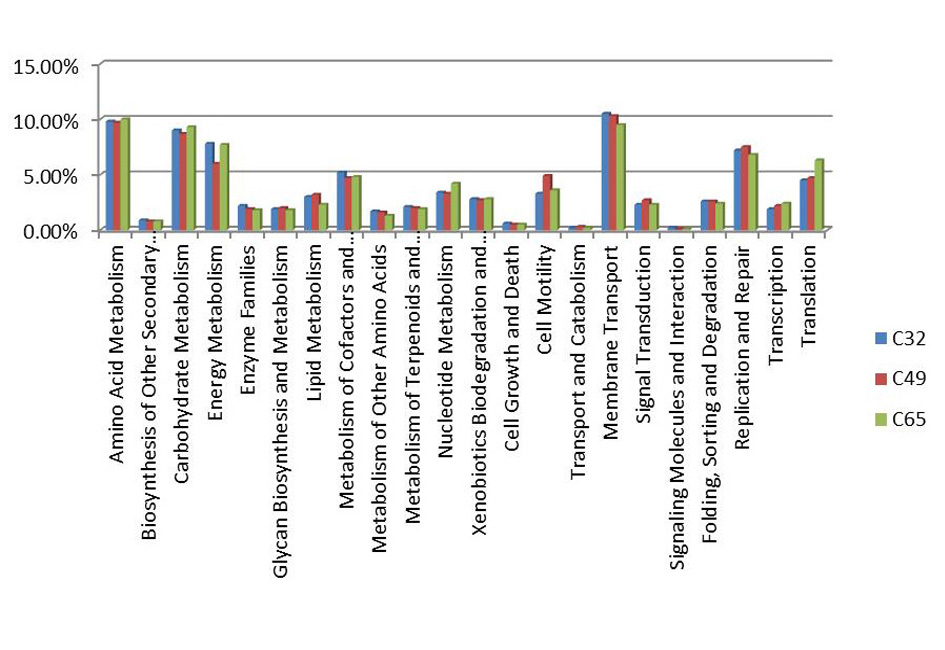

Supplement: Supplementary Figure S4 — Relative abundance of gene counts in the C32, C49 and C65 microbialite samples from Ciocaia for selected functional KEGG pathways inferred from 16S rRNA gene data using PICRUSt (Langille et al., 2013). [file Image4.JPEG]
