# Supplementary material for: Structure, mineralogy, and microbial diversity of geothermal spring microbialites associated with a deep oil drilling in Romania
Source: Front Microbiol. 2015 Mar 30;6:253. doi: 10.3389/fmicb.2015.00253 (PMC4378309; doi:10.3389/fmicb.2015.00253)
Supplement: Supplementary file 5 [file DataSheet1.DOCX]

Supporting information for methods – primers used

**Structure, mineralogy and microbial diversity of geothermal spring microbialites associated with a deep oil drilling in Romania**

Cristian Coman^*^, Cecilia M. Chiriac, Michael S. Robeson, Corina Ionescu, Nicolae Dragos, Lucian Barbu-Tudoran, Adrian-Stefan Andrei, Horia L. Banciu, Cosmin Sicora, Mircea Podar

*cristian.coman@icbcluj.ro

**Primers used for Quantitative real-time PCR amplification**

931F *Archaea* AGGAATTGGCGGGGGAGCA (Einen et al., 2008);

M1100R *Archaea* BGGGTCTCGCTCGTTRCC (Einen et al., 2008);

338F *Bacteria* ACTCCTACGGGAGGCAGCAG (Lane, 1991);

518R *Bacteria* ATTACCGCGGCTGCTGG (Muyzer et al., 1993).

**Primers used for amplification of V4 region for paired-end Illumina sequencing** (Lundberg et al., 2013).

| **Sequence Description** | | **Sequence** | **Coverage** |
| --- | --- | --- | --- |
| Diversity generation primers | 515F_f1 | GCCTCCCTCGCGCCATCAGAGATGTGTATAAGAGACAGNNNNNNNNGAGTGCCAGCMGCCGCGGTAA | Bacteria+Euryarchaeota |
|  | 515F_f2 | GCCTCCCTCGCGCCATCAGAGATGTGTATAAGAGACAGNNNNTNNNNGAGTGCCAGCMGCCGCGGTAA |  |
|  | 515F_f3 | GCCTCCCTCGCGCCATCAGAGATGTGTATAAGAGACAGNNNNCTNNNNGAGTGCCAGCMGCCGCGGTAA |  |
|  | 515F_f4 | GCCTCCCTCGCGCCATCAGAGATGTGTATAAGAGACAGNNNNACTNNNNGAGTGCCAGCMGCCGCGGTAA |  |
|  | 515F_f1TM7 | GCCTCCCTCGCGCCATCAGAGATGTGTATAAGAGACAGNNNNNNNNGAGTGCCAGCMGCCGCGGTCA | TM7 |
|  | 515F_f4Arc | GCCTCCCTCGCGCCATCAGAGATGTGTATAAGAGACAGNNNNACTNNNNGAGTGKCAGCMGCCGCGGTAA | mainly Crenarchaeota |
|  | 806R_f1 | GTGACTGGAGTTCAGACGTGTGCTCTTCCGATCTNNNNNACGGACTACHVGGGTWTCTAAT | universal reverse |
|  | 806R_f2 | GTGACTGGAGTTCAGACGTGTGCTCTTCCGATCTNNTNNNACGGACTACHVGGGTWTCTAAT |  |
|  | 806R_f3 | GTGACTGGAGTTCAGACGTGTGCTCTTCCGATCTNNCTNNNACGGACTACHVGGGTWTCTAAT |  |
|  | 806R_f4 | GTGACTGGAGTTCAGACGTGTGCTCTTCCGATCTNNACTNNNACGGACTACHVGGGTWTCTAAT |  |
| Barcoded primers | PCR_F1 | AATGATACGGCGACCACCGAGATCTACACGCCTCCCTCGCGCCATCAGAGATGTG |  |
|  | 806rcbc1567 | CAAGCAGAAGACGGCATACGAGATGACTAGTCAGCTGTGACTGGAGTTCAGACGTGTGCTC | Specific tag for C32 |
|  | 806rcbc1680 | CAAGCAGAAGACGGCATACGAGATCTCTGAGGTAACGTGACTGGAGTTCAGACGTGTGCTC | Specific tag for C49 |
|  | 806rcbc1729 | CAAGCAGAAGACGGCATACGAGATTCACTGCTAGGAGTGACTGGAGTTCAGACGTGTGCTC | Specific tag for C65 |

References

Einen J, Thorseth IH, Ovreås L. Enumeration of Archaea and Bacteria in seafloor basalt using real-time quantitative PCR and fluorescence microscopy. *FEMS Microbiol Lett* (2008) **282**:182-187.

Lane DJ. “16S/23S rRNA sequencing”. In: Stackebrandt E, Goodfellow M, editors. *Nucleic acid techniques in bacterial systematics*. United Kingdom: John Wiley & Sons Ltd., (1991) p. 115–175.

Lundberg DS, Yourstone S, Mieczkowski P, Jones CD, Dangl JL. Practical innovations for high-throughput amplicon sequencing. *Nat Methods* (2013) **10**:999–1002.

Muyzer G, Waal EC, Uitterlinden AG. Profiling of complex microbial populations by denaturing gradient gel electrophoresis analysis of polymerase chain reaction-amplified genes coding for 16S rRNA. *Appl Environ Microbiol* (1993) **59**:695-700.
