# Supplementary material for: Structure, mineralogy, and microbial diversity of geothermal spring microbialites associated with a deep oil drilling in Romania
Source: Front Microbiol. 2015 Mar 30;6:253. doi: 10.3389/fmicb.2015.00253 (PMC4378309; doi:10.3389/fmicb.2015.00253)
Supplement: Supplementary file 6 [file DataSheet2.ZIP › ciocaia_mineral_deposit---ssu---krona----Total---sim_97---tax_silva---td_20.html]

Javascript must be enabled to view this page.

members
magnitude

ciocaia\_mineral\_deposit---ssu---krona---C32----Total---sim\_97---tax\_silva---td\_20
ciocaia\_mineral\_deposit---ssu---krona---C49----Total---sim\_97---tax\_silva---td\_20
ciocaia\_mineral\_deposit---ssu---krona---C65----Total---sim\_97---tax\_silva---td\_20

6766512551499086

3

3

3

3

3

3

3

3

6492812230656902

318

318

318

318

318

1915201

3

3

4

22055211

1716

1

6

17

220351

172091

172091

25881

78

1141

6

3763

4826

4826

4815

11

35

442

442

442

682842577

682842577

682842577

682842577

5

682842572

1945111271730346

1

5787400151

177765

33

4

29

436

413

6

17

1

1

2

1542

10

132

131

115135

11515

3

1

1

69

10

5

28

20

20

6

6

3191

17

22

12

10

2

341

1

34

234

9

16

117

92

3206

317

24

102

184

36

2

4

3

169

22

9778

7578

3

7575

22

22

3014380138

3014380138

1266194135

10403

6

3

97

59

251

186

5

76171

17

1112

1064412410

29168

21

21

13

13

41

41

6647

61

200

200

1013289

1

1

10

10

1013278

1013278

3

3

3

30922

285

2422

24

22

97

11

11

86

86

152

73

17

30

26

79

42

37

111827

111827

111827

6

1343

1343

211

11

2

1132

632

5

873082701171

17

22

2

2

2

65

437

437

89

2

2

344

24

24

24

323430411

833

833

124428661

124428661

191142

31142

3

148

4

5

21

1

1

2

2

633

633

633

65017

4951

6

6

1551

1

24

1

130

1

1

1

9783067645

3

37

576306759

1

1

576306739

302136

9

1

292136

54

54

18283

10283

10283

8

5

3

122

122

122

5538897834

1892895734

10

1688

1711895726

364621

17

351621

113

9

9

9

38452548830011

2611035

285

85

2

142

142

1

1

248728

6

135

97417

15

1483862

1483862

1448

35741

121

3

3

3

23332451629976

23332451629976

1

7053661115

22631914928861

5

14

1

4

9

20121579

20121579

20121579

20121579

65

94058

748

342

419421

334148

300148

300148

300148

300148

13121620

13121620

13121620

13121620

13121620

3858963101

3858963101

3101

3101

3101

385896

385596

385596

3

3

251

230

144

144

138

6

86

21

21

3

3

18

6

2108

416561627

9

1254510

1254510

4

1

25

44

76

6

7

93

93

8

18921

54

54

54

703

71

71

547

56

85

319

4

83

85

84

1

11351

7

219

156

5

1

1

748

340

210

145848112

145848112

141734910

1331

3

131

28128

4128

11

1

1

11815

11815

2015

2015

98

8

2

16

72

5

88

26

1

1

1

1

1

22

22

1

16

16

16

2

2

2

3

4521

4521

451

451

451

11

2055644532

2055644532

2055644532

2055644532

55955

200971206

4102371

28

24

15

73173

73173

3

73170

73411364

73411364

73411364

73411364

3401364

1

7

1103

1103

1103

17

13

4

33

33

176233

1

571

5

4

1

1

521

521

80621

2

2

1

1

25321

311

31

1

11

3

3

5

25

16

9

1051

1051

109

109

551

547

547

4

4

5841

5841

264

175

175

4

4

1411

66

751

307

8

8

8

299

299

299

1

7

7

7

7

2592153

2592153

2592153

333

333

259181

242181

17

1

1

1

1483228

97

278227

278227

278227

3

12

23

132

66227

15

27

10081

3

3

986

986

15

971

1

16

98

2

6227252512676

686247412632

1934511233

1934311227

201

5

729

1524410753

8852

230

42157

6

6

1

1

1

117134

66621121265

6367

14

1531

1

3435

1243145

42

49

101

93

8

7

1343

1343

460

460

8

8

248890768

46

416

230290

195

259690

928

35678

3397127

2

2487126

32

1

7

41

2

2

21126365

11

17786

278

2044

4

40114

40114

61

61

16

5505244

89

6

6

83

3

8

5497235

10

10

1

1

5

5

5482221

2

28

5454219

94

9

4

49

49

49

20

20

20

20

3789100531

13

13

32

3

7

1

34

34

34

34

1601105

1601105

1601105

1601105

22

22

22

1

21

178689427

178689427

178689427

1

572

17863171

4

345

33

312

312

312

121

13993

25

72

72

72

72

112

83824

382

382

382

84

84

4

8

8599

260327946752

13141435432

4

4

4

4

4

1441

1441

1419

22

22

22

13139635391

9510

64

64

1

63

3110

3110

3110

759865

759865

2

739865

12101

201547

9255

1

212

9

8

164

1

15

6108

5620135265

5620135265

5620135265

246

5415535265

39

39

39

39

12

12

12

12

2

2

1

1

1
